# α-Lipoic Acid, with or Without Ambrisentan, Attenuates Experimental Pulmonary Arterial Hypertension: Associations with AMPK/mTOR/NF-κB Signaling and Kallistatin

**DOI:** 10.3390/biom16101432

**Published:** 2026-10-02

**Authors:** Seyhan Ayik, Mustafa Huz, Zeynep Ulutas, Mehmet Ertugrul Balkar, Kevser Tanbek, Rumeysa Sonmez, Ruveyda Ummugulsum Unal, Eylem Karadag, Seymanur Baydak, Lokman Hekim Tanriverdi, Onural Ozhan

**Affiliations:** 1 Department of Medical Pharmacology, Faculty of Medicine, İnönü University, Malatya 44280, Türkiye; mertugrul.balkar@inonu.edu.tr (M.E.B.); lokmanhekim.tanriverdi@inonu.edu.tr (L.H.T.); onural.ozhan@inonu.edu.tr (O.O.); 2 Department of Medical Pathology, Faculty of Medicine, İnönü University, Malatya 44280, Türkiye; mustafa.huz@inonu.edu.tr; 3 Department of Cardiology, Faculty of Medicine, İnönü University, Malatya 44280, Türkiye; zeynep.ulutas@inonu.edu.tr; 4 Department of Physiology, Faculty of Medicine, İnönü University, Malatya 44280, Türkiye; kevser.tanbek@inonu.edu.tr; 5 Department of Medical Pharmacology, Institute of Health Sciences, İnönü University, Malatya 44280, Türkiye; 38244201001@ogr.inonu.edu.tr (R.S.); 38244201002@ogr.inonu.edu.tr (R.U.U.); 38244201004@ogr.inonu.edu.tr (E.K.); 38244201003@ogr.inonu.edu.tr (S.B.)

**Keywords:** Alpha-lipoic acid, ambrisentan, AMPK, kallistatin, mTOR, pulmonary arterial hypertension

## Abstract

Background: Pulmonary arterial hypertension (PAH) is characterized by progressive pulmonary vascular remodeling driven by excessive cellular proliferation and inflammation. Targeting both vasoconstriction and vascular remodeling represents a key therapeutic strategy. Alpha-lipoic acid (α-LA), a metabolic and anti-inflammatory modulator, is associated with modulation of AMPK/mTOR/NF-κB signaling, whereas ambrisentan (AMB) is an endothelin receptor antagonist with established vasodilatory effects. We investigated the beneficial effects of α-LA and AMB, administered alone or in combination, in a monocrotaline (MCT)-induced PAH rat model. Methods: Male rats were randomly assigned to control, MCT, α-LA, AMB, and α-LA + AMB groups. PAH was induced by a single subcutaneous injection of monocrotaline (60 mg/kg). Treatments were administered between days 22 and 35. Echocardiographic measurements were obtained, and histopathological, immunohistochemical, and biochemical analyses were performed in lung and cardiac tissues and in serum. Sample sizes analyzed per outcome were: echocardiography (Control: *n* = 10, MCT: *n* = 10, α-LA: *n* = 8, AMB: *n* = 9, α-LA + AMB: *n* = 10), histology (*n* = 10/group), RVSP (Control: *n* = 7, MCT: *n* = 8, α-LA: *n* = 8, AMB: *n* = 7, α-LA + AMB: *n* = 9), Fulton index (Control: *n* = 10, MCT: *n* = 10, α-LA: *n* = 12, AMB: *n* = 9, α-LA + AMB: *n* = 10), and ELISA (*n* = 8/group). Statistical analyses were performed using a web-based statistical software, applying one-way ANOVA or the Kruskal–Wallis test according to data distribution. Results were considered statistically significant at *p* < 0.05. Results: MCT administration caused pulmonary hypertension and right ventricular remodeling, evidenced by increased RVSP, Fulton index, medial wall thickness, cardiomyocyte diameter, PCNA, α-SMA, p-mTOR, and NF-κB, together with reduced PAT, TAPSE, kallistatin, and p-AMPK levels. α-LA and AMB monotherapy improved right ventricular function and inflammation. Combination therapy produced hemodynamic and structural improvements relative to MCT that were generally comparable to, rather than significantly greater than, either monotherapy, and showed attenuated antiproliferative effects, with higher cardiomyocyte diameter and PCNA levels than either monotherapy alone. All treatments suppressed mTOR activation and the NF-κB increase, accompanied by increased serum kallistatin levels, whereas a significant increase in p-AMPK relative to MCT was observed only with AMB monotherapy. Conclusions: These findings demonstrate that α-LA exerts antiproliferative, anti-remodeling, and anti-inflammatory effects in experimental PAH, suggesting that the AMPK/mTOR/NF-κB signaling pathway and kallistatin may be potential therapeutic targets in pulmonary vascular disease.

## 1. Introduction

Pulmonary hypertension (PH) is a complex medical condition involving multiple clinical entities, frequently associated with various cardiovascular and respiratory diseases. It is characterized as a hemodynamic state with a mean pulmonary arterial pressure (mPAP) exceeding 20 mmHg, measured via right heart catheterization. PH is classified into several subgroups based on clinical and hemodynamic features, with pulmonary arterial hypertension (PAH) included in Group 1, which is notably distinguished by primary involvement of the pulmonary vascular bed [1]. Because the monocrotaline (MCT) model used in this study reproduces pulmonary hypertension with a substantial inflammatory/toxic vascular-injury component rather than the full spectrum of Group 1 PAH pathobiology, we use “pulmonary hypertension (PH)” when referring to the general hemodynamic condition or to the MCT model itself, and reserve “pulmonary arterial hypertension (PAH)” for the human Group 1 disease construct and its translational context, consistently throughout this manuscript.

PAH is a severe cardiopulmonary disease characterized by progressive narrowing of small pulmonary arteries and arterioles [2]. Increased pulmonary arterial pressure and vascular resistance can lead to right ventricular hypertrophy (RVH) and right heart failure [3]. In the pathogenesis of PAH, vasoconstriction is not the only mechanism; multidimensional processes, including cellular proliferation, inflammation, oxidative stress, and metabolic reprogramming in the pulmonary vessel wall, also play a role [4]. In particular, vascular remodeling, driven by abnormal proliferation and migration of pulmonary artery smooth muscle cells, narrows the vascular lumen and permanently elevates pulmonary vascular resistance (PVR) [5]. Therefore, PAH is currently considered a proliferative and inflammatory vasculopathy [4,5]. Endothelin receptor antagonists (ERAs) that block endothelin-1’s vasoconstrictive and mitogenic effects provide significant clinical benefits [6]. However, ERAs do not fully target proliferative pathways or stop disease progression. Long-term side effects and limited efficacy highlight the need for alternative or combined treatments [7].

Recent studies have focused on the relationship between cellular energy metabolism and proliferation in PAH treatment and pathology [8]. As a key regulator of cellular energy homeostasis, adenosine monophosphate-activated protein kinase (AMPK) is activated by a decrease in the intracellular ATP/AMP ratio [9]. Activated AMPK negatively regulates the mammalian target of rapamycin (mTOR) pathway, which is directly associated with increased PASMC proliferation and vascular remodeling in PAH [10]. mTOR stimulates anabolic processes, including protein synthesis, to support cell proliferation and growth, and, as recent data indicate, functions as a key regulator of mitochondrial energy production [11]. Additionally, nuclear factor kappa B (NF-κB) signaling, which plays a central role in PAH pathogenesis, enhances inflammatory responses by increasing the expression of pro-inflammatory cytokines (such as IL-1β, IL-6, and monocyte chemoattractant protein-1 [MCP-1]) [12].

Alpha-lipoic acid (α-LA) is a natural short-chain fatty acid that serves as a cofactor in multienzyme complexes that catalyze the oxidative decarboxylation of α-keto acids such as pyruvate and α-ketoglutarate. α-LA is of interest as a potent antioxidant and metabolic regulator; it scavenges free radicals, suppresses inflammation, and reduces oxidative stress [13]. α-LA has been shown to activate AMPK, thereby limiting cell proliferation by suppressing mTOR signaling. Zhang et al. reported that α-LA reduced oxidative stress and improved nerve function in diabetic peripheral neuropathy by activating the AMPK/Nrf2 pathway [14]. Similarly, Jeon et al. demonstrated that α-LA suppressed thyroid cancer cell proliferation by downregulating mTOR signaling via AMPK activation [15]. These findings indicate that α-LA may serve as a potential therapeutic agent by supporting vascular and cellular homeostasis through both metabolic and inflammatory mechanisms [16].

Ambrisentan is an established endothelin receptor antagonist with proven efficacy in PAH, although its therapeutic effects have been evaluated mainly in terms of hemodynamic and clinical outcomes rather than in combination with α-lipoic acid [17,18,19]. α-Lipoic acid is a well-known antioxidant and anti-inflammatory compound, but its potential in experimental PAH remains insufficiently explored [20,21,22,23,24]. In addition, the AMPK/mTOR/NF-κB axis plays a central role in pulmonary vascular remodeling, while kallistatin has emerged as a vasoprotective mediator associated with pulmonary vascular injury; however, these mechanisms have not been investigated in the context of combined α-lipoic acid and ambrisentan treatment in experimental PAH [25,26,27,28,29,30,31,32]. Therefore, this study aimed to evaluate the effects of α-lipoic acid and ambrisentan, administered alone and in combination, in a monocrotaline-induced, in vivo PAH rat model, while examining their associations with the AMPK/mTOR/NF-κB signaling and kallistatin.

We hypothesized that α-LA and AMB, administered alone or in combination, would attenuate MCT-induced PAH by modulating AMPK/mTOR/NF-κB signaling and kallistatin levels, with the primary endpoints being RVSP and echocardiographic indices of right ventricular function (PAT, RV/LV diameter ratio, and TAPSE). Because this study was designed and powered as an exploratory comparison against MCT rather than as a formal superiority trial, the comparison of combination therapy against each monotherapy was treated as a secondary, exploratory objective rather than a prespecified primary hypothesis.

## 2. Materials and Methods

### 2.1. Experimental Design

This study was approved by the Local Ethics Committee of Animal Experiments at İnönü University (Approval No. 2023/7-6 and Approval date: 3 August 2023). Fifty-eight male Wistar Albino rats, 3 months old and weighing an average of 300 g, were used in our study. The animals were obtained from the Laboratory Animal Production and Research Center at İnönü University. All rats were naive animals with no prior experimental procedures and had a healthy immune status. The rats were fed fresh tap water and rat chow ad libitum. The rats were housed under constant temperature (23 ± 1 °C) and humidity (45–50%) conditions, with a 12-h light/12-h dark cycle. Prior to the initiation of the experimental procedures, all animals were allowed an acclimatization period of 7 days to adapt to the laboratory environment and housing conditions. No additional environmental enrichment (such as nesting material or plastic tunnels) was introduced to prevent potential confounding factors in vascular response studies. In the present study, the National Institutes of Health’s Animal Research guidelines and ARRIVE 2.0 guidelines were strictly followed.

Randomization and Blinding: The experimental unit was an individual rat. Animals were randomly assigned to five groups using a computer-generated random number sequence (www.randomizer.org). To minimize confounders, cage rack positions and the daily order of treatments and procedures were systematically rotated. Group allocation was concealed during drug administration using coded solutions. All outcome evaluations (echocardiography, RVSP, and histopathology/IHC) and statistical analyses were performed by investigators blinded to the treatment groups.

The rats were assigned to five groups: Control (*n* = 10), Monocrotaline (MCT, *n* = 12), α-LA (*n* = 12), AMB (*n* = 12), and α-LA + AMB (*n* = 12). All animals that met the predefined study characteristics (male Wistar Albino rats, 3 months of age, with an average body weight of approximately 300 g) were included in the study.

In accordance with ARRIVE 2.0 guidelines regarding sample size reporting and data exclusion, sample sizes for each experimental modality differed due to technical feasibility, quality-control criteria (e.g., poor signal-to-noise ratio in echocardiography, signal loss during hemodynamics), and tissue availability constraints. Specific exclusion criteria were defined a priori based on quality standards: (1) hemodynamics (RVSP): cannulation artifacts or unstable baseline blood pressure traces; (2) echocardiography: inadequate acoustic window or signal artifact; and (3) ELISA: insufficient serum/tissue volume or hemolyzed samples. The precise number of analyzed animals (n) per group for each parameter was as follows:

(1) Echocardiography: Control (*n* = 10), MCT (*n* = 10), α-LA (*n* = 8), AMB (*n* = 9), α-LA + AMB (*n* = 10);
(2) Histopathology and Immunohistochemistry: Control (*n* = 10), MCT (*n* = 10), α-LA (*n* = 10), AMB (*n* = 10), α-LA + AMB (*n* = 10);
(3) Right Ventricular Systolic Pressure (RVSP): Control (*n* = 7), MCT (*n* = 8), α-LA (*n* = 8), AMB (*n* = 7), α-LA + AMB (*n* = 9);
(4) Fulton Index: Control (*n* = 10), MCT (*n* = 10), α-LA (*n* = 12), AMB (*n* = 9), α-LA + AMB (*n* = 10);
(5) ELISA Analyses (p-mTOR, mTOR, p-AMPK, Total AMPK, Kallistatin, NF-κB): Control (*n* = 8), MCT (*n* = 8), α-LA (*n* = 8), AMB (*n* = 8), α-LA + AMB (*n* = 8).

The PAH model was induced by subcutaneous administration of 60 mg/kg MCT on day 1. The treatment groups received α-LA (100 mg/kg, s.c.) and/or AMB (0.2 mg/kg/day, per os) between days 22 and 35. This treatment window was selected based on previously reported time-course data indicating that MCT-induced hemodynamic and structural changes are consistently established by approximately day 21–28 post-injection, thereby modeling a therapeutic rather than a preventive intervention [33,34].

Animals were monitored daily for general health, body weight, and signs of pain or distress. Predefined humane endpoints were established for early euthanasia in cases of severe distress or >20% weight loss. No animals reached humane endpoints, and no unexpected adverse events occurred during the study.

**Figure 1:**
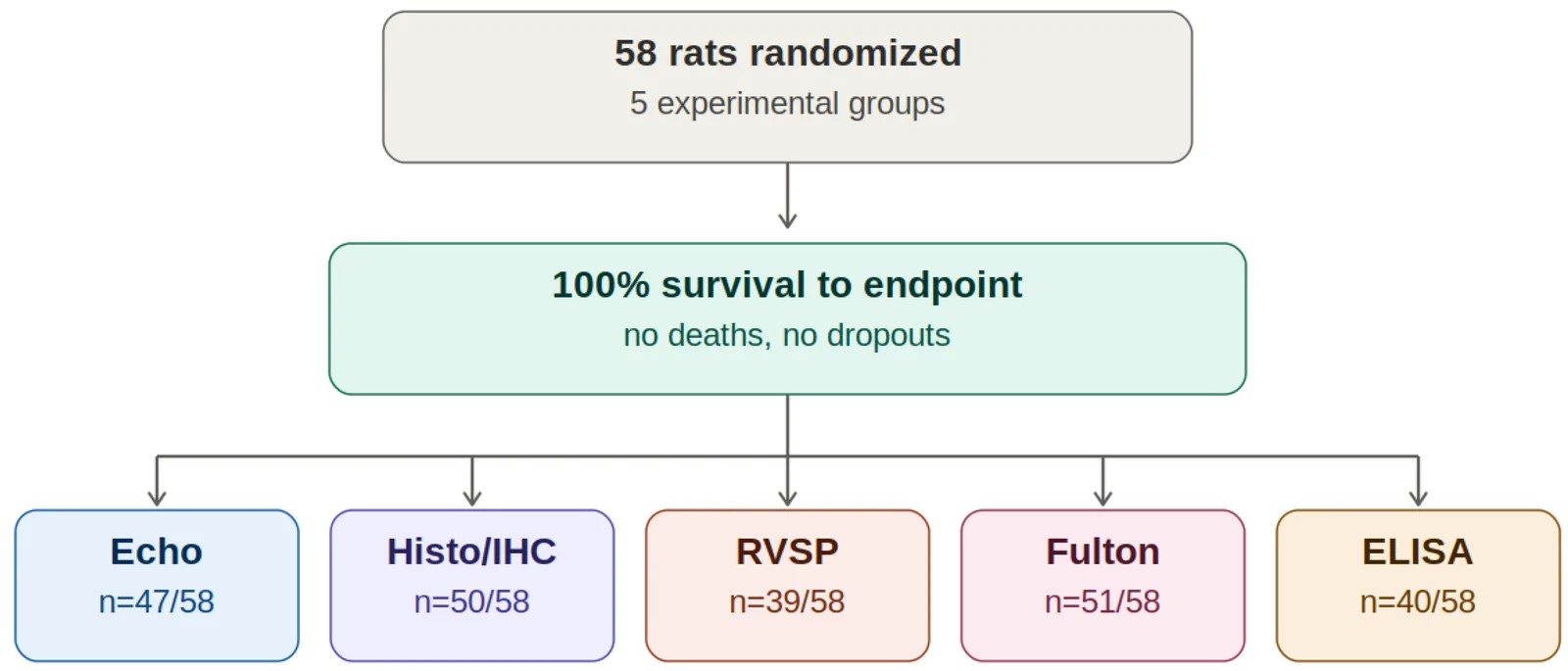
Flowchart summarizing animal randomization, survival to study endpoint, and the number of animals analyzed per outcome measure (of 58 total).

A total of 58 rats were randomized into five experimental groups (Control, *n* = 10; MCT, *n* = 12; α-LA, *n* = 12; AMB, *n* = 12; α-LA + AMB, *n* = 12). All 58 animals survived to the predefined study endpoint (100% survival in every group); consequently, no animals were excluded from the study due to death or premature euthanasia. The number of animals analyzed for each outcome measure varied because outcome-specific, a priori technical and quality-control criteria were applied, not because of animal loss. For echocardiography, 47 of 58 animals (81.0%) were analyzed; 11 were excluded due to inadequate acoustic window and/or image artifacts. For histopathological/immunohistochemical analysis, 50 of 58 animals (86.2%) were analyzed; 8 were excluded because of inadequate tissue preservation, sectioning artifacts, and/or poor staining quality. For right ventricular systolic pressure (RVSP), 39 of 58 animals (67.2%) were analyzed; 19 were excluded due to cannulation-related technical failure and/or unstable pressure tracing. For the Fulton index, 51 of 58 animals (87.9%) were analyzed; 7 were excluded because of technical problems during ventricular separation or unreliable ventricular weight measurements. For ELISA-based biomarker quantification, 40 of 58 animals (69.0%) were analyzed; 18 were excluded due to sample quality issues, technical assay problems, out-of-range values, and/or inconsistent duplicate measurements. All exclusion criteria were predefined and applied uniformly across groups. Animal allocation, survival, and outcome-specific exclusions are summarized in Figure 1 and Table 1 and Table 2.

At the end of the experiment, all rats were anesthetized by intraperitoneal administration of ketamine hydrochloride (75 mg/kg) and xylazine (8 mg/kg). Adequate depth of anesthesia was ensured before all procedures. Right ventricular systolic pressure [RVSP] measurement (via jugular vein cannulation) was performed under deep anesthesia in all groups. Blood samples were then collected via intracardiac puncture using a sterile syringe and euthanasia was performed by exsanguination through the same procedure under deep anesthesia. Serum samples were obtained by centrifugation of blood samples and stored at −80 ◦C until assayed. Hearts and lungs were fixed in 10% formaldehyde for histopathological evaluation.

The doses of MCT (Sigma-Aldrich LLC, St. Louis, MO, USA; CAS No. 315-22-0), α-LA (Apollo Scientific Ltd, Stockport, UK; CAS No. 1200-22-2), and AMB (Volimra^®^ 10 mg film-coated tablet; Deva Holding JSC, Istanbul, Türkiye) were determined based on earlier reports [13,33,34].

### 2.2. Echocardiography

Echocardiographic assessments were performed by an investigator blinded to group allocation. Statistical analysis of the echocardiographic data was performed independently by a second investigator, also blinded to group allocation. Prior to imaging, rats were anesthetized with ketamine hydrochloride (75 mg/kg; Ketalar^®^, Pfizer, İstanbul, Türkiye) and xylazine (8 mg/kg; Rompun^®^, Bayer, İstanbul, Türkiye), administered intraperitoneally, and their chests were shaved. Rats were examined using transthoracic echocardiography with a 10S-RS sector probe (GE Parallel Design Inc., Phoenix, AZ, USA) on a Vivid S6 N ultrasound system (GE Vingmed Ultrasound AS, Horten, Norway ), following previously reported techniques [35]. For the transthoracic echocardiography, both two-dimensional (2D) and M-mode techniques were used, in addition to Doppler measurements. The animals were evaluated in the left lateral decubitus position. PAT was measured from the parasternal short-axis view, the RV/LV diameter ratio was measured in the apical four-chamber view, and TAPSE was measured in the RV-focused apical four-chamber view. Heart rate was measured during echocardiography, calculated from the average interval between five consecutive mitral E-waves. Echocardiographic analyses of PAT, TAPSE, RV/LV diameter ratio, and heart rate are presented in Section 3.1.

### 2.3. Right Ventricular Systolic Pressure Measurement

Right ventricular systolic pressure (RVSP) was measured via a slightly curved-tip catheter (umbilical catheter) inserted into the right jugular vein, connected to a pressure transducer and BIOPAC MP-100 Data Acquisition System with AcqKnowledge software version 3.7.2 (BIOPAC Systems, Inc., Santa Barbara, CA, USA). The pressure transducer was calibrated using a two-point calibration procedure prior to each measurement, with atmospheric pressure defined as 0 mmHg and a known reference pressure applied; the transducer was positioned at heart level, and the zero-pressure check was repeated before each recording. The curved catheter tip guided its passage through the right atrium into the right ventricle. Catheter positioning was confirmed and guided in real time via the pressure waveform: the catheter was advanced gradually until a characteristic right ventricular pressure waveform (identified by its characteristic morphology, i.e., a rapid systolic upstroke followed by a low diastolic plateau; transient negative diastolic deflections attributable to respiratory variation, catheter movement, or transducer zeroing were occasionally observed and did not affect the systolic peak values used to calculate RVSP) was obtained. Five consecutive cycles with consistent waveform morphology were averaged for each measurement. Waveforms not meeting these criteria were considered artifactual and were excluded.

### 2.4. Fulton Index

Hearts were fixed in 10% formalin, then carefully sectioned transversely, with the atria gently separated from the ventricles. Next, the right ventricular (RV) free wall was carefully removed. To assess whether RVH is present, the Fulton index was calculated by dividing the weight of the RV free wall by the combined weight of the left ventricle (LV) and interventricular septum (IVS) [33].

### 2.5. Histopathology

Following the sacrifice of the subjects, the hearts and lungs were excised, and tissue samples were collected for histopathological examination. These samples were immediately immersed in 10% buffered formalin and fixed for 12 h. Tissue processing was conducted using a fully automated closed-system device (Shandon Excelsior ES; Thermo Fisher Scientific, Runcorn, UK), employing sequential alcohol, xylene, and paraffin series, completing the entire process within 12 h. For histopathological and immunohistochemical (IHC) analyses, four-micrometer-thick sections were prepared from formalin-fixed, paraffin-embedded (FFPE) specimens using a rotary microtome (RM2255; Leica Biosystems, Nussloch, Germany). Histological evaluation involved hematoxylin and eosin (H&E) staining, as well as Masson’s trichrome staining. For immunohistochemistry, sections were incubated with Anti-α-SMA Antibody (Thermo Fisher Scientific Inc., Waltham, MA, USA, clone17H19L35) at a dilution of 1:100 and Anti-PCNA Antibody (Atlas Antibodies AB., Bromma, Sweden Catalog no: HPA030521) at a dilution of 1:500, each for 30 min. Imaging was performed using an automated IHC platform (Dako Omnis; Dako/Agilent Technologies, Glostrup, Denmark) with the Dako EnVision FLEX+ detection system(Agilent Technologies/Dako, Glostrup, Denmark; Cat. No. K8000) and DAP chromogen according to standard histological and immunohistochemical procedures [36,37].

Histopathological evaluation of pulmonary vascular lesions and inflammation was performed according to established scoring systems [38,39] (see Table 3 and Table 4 for detailed criteria). For immunohistochemical assessment, a semi-quantitative H-score was calculated by multiplying the staining intensity (0–3) by the extent of staining (0–3). This H-score (range 0–9, dimensionless) was applied to quantify both PCNA and α-SMA expression, the latter assessed specifically within the tunica media of pulmonary arteries.

Both the histopathological scoring (vascular lesion and pulmonary inflammation scores) and the semi-quantitative immunohistochemical H-score analysis (PCNA and α-SMA) were performed by a pathologist and image analysts, respectively, who were blinded to treatment group allocation throughout the scoring process; group identity was concealed via coded slide labels and was not revealed until all quantitative data had been finalized.

**Figure 2:**
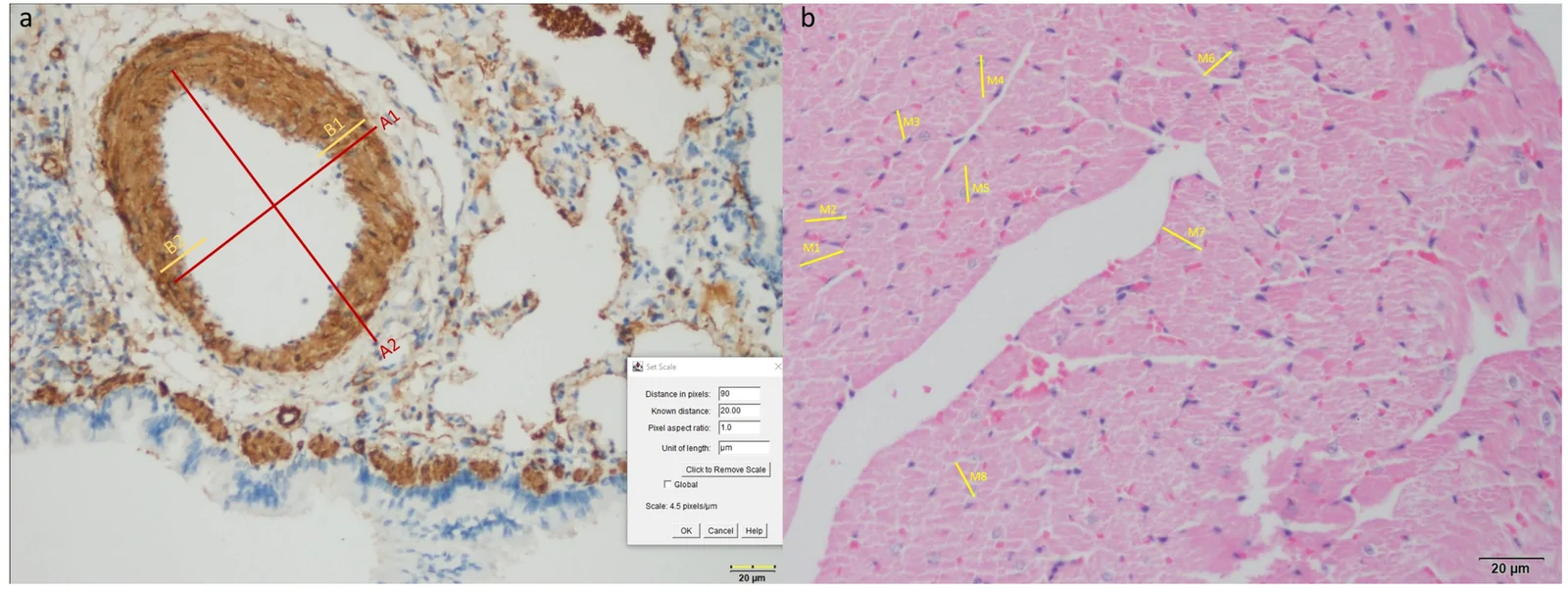
Representative images illustrating the morphometric measurement methods used in this study. (**a**) Tunica media thickness measurement in a pulmonary artery cross-section (α-SMA immunostaining). A1 and A2 indicate the external vessel diameter measured along two perpendicular axes; B1 and B2 indicate the corresponding tunica media (wall) thickness measured along the same axes. (**b**) Cardiomyocyte diameter measurement in a transversely sectioned cardiac muscle fiber (H&E staining). Yellow lines (M1–M7) indicate representative transnuclear width measurements, each drawn perpendicular to the fiber’s long axis at the level of a clearly visible, centrally located nucleus. Scale bars, 20 µm.

Morphometric measurements were performed by an observer blinded to group allocation. For each animal, three non-serial lung sections were evaluated, and ten small-to-medium pulmonary arteries per section (30 vessels per animal in total) were selected using a systematic random sampling approach within the predefined external diameter range. Tunica media thickness was quantified using the formula: α = (B1 + B2)/(A1 + A2), where A1 and A2 represent the external vessel diameter measured along two perpendicular axes (vertical and horizontal), and B1 and B2 represent the corresponding tunica media (wall) thickness measured along the same two axes (Figure 2a). Measurements were performed along two perpendicular axes to minimize the potential bias introduced by oblique/tangential sectioning of the vessel wall, which can artificially distort wall thickness when assessed along a single axis. For each vessel, the ratio of mean medial thickness to mean external diameter was calculated; values from the 30 vessels per animal were averaged to yield a single value per animal, which served as the unit of statistical analysis.

This tunica-media index is expressed as a dimensionless ratio of medial (wall) thickness to external vessel diameter (equivalent to percentage medial wall thickness when multiplied by 100), conceptually analogous to established morphometric measures of pulmonary vascular remodeling used in the PAH literature.

Cardiomyocyte diameter was measured as the transnuclear width at the level of a clearly visible, centrally located nucleus, in transversely sectioned fibers exhibiting a round-to-polygonal cross-sectional profile, consistent with established criteria for cardiomyocyte size quantification in H&E-stained sections (Figure 2b) [40]. All morphometric analyses were conducted using ImageJ software (version 1.54f; National Institutes of Health, Bethesda, MD, USA; https://imagej.net/ij/) [41].

The vascular lesion score (Table 3) was calculated as the sum of the eight individual component scores, yielding a composite score ranging from 0 to 14. The pulmonary inflammation score (Table 4) represents a single ordinal grade (range 0–4, with a score of 0 assigned when no inflammatory infiltrate was present ), corresponding to the most advanced or predominant inflammatory pattern identified in a given section, rather than the sum of independent, co-occurring findings.

### 2.6. Enzyme-Linked Immunosorbent Assay (ELISA)

Blood samples were collected into tubes without anticoagulant, allowed to clot at +4 °C for 1 h, and then centrifuged at 1800× *g* for 10 min at 4 °C to separate the serum. Serum samples were aliquoted and stored at −80 °C until analysis. Lung tissue samples (0.1 g) were homogenized on ice in 1 mL of ice-cold phosphate-buffered saline (PBS, pH 7.4) supplemented with a protease inhibitor cocktail. The homogenates were centrifuged at 16,000× *g* for 15 min at 4 °C in a refrigerated centrifuge. The resulting supernatants were collected, aliquoted, and stored at −80 °C until further processing. Total protein concentrations of the lung homogenate supernatants were determined using the Bradford assay method [42] with bovine serum albumin (BSA) as the standard, and total protein levels were used to normalize tissue biomarker concentrations. All ELISA analyses were performed strictly according to the manufacturers’ instructions. Absorbance was read at 450 nm using a microplate reader (Synergy LX multi-mode reader; BioTek, Agilent Technologies, Santa Clara, CA, USA).

Serum Kallistatin levels were measured using a rat-specific ELISA kit (Cat. No. SL1892Ra, Sunlong Biotech Co., Ltd., Hangzhou, China; detection range: 0.15–8 ng/mL, sensitivity: 0.05 ng/mL). Lung tissue levels of p-AMPK (Cat. No. E0436Ra, Bioassay Technology Laboratory (BT LAB), Shanghai, China; detection range: 0.5–200 U/L, sensitivity: 0.25 U/L), total AMPK (Cat. No. E2094Ra, BT LAB; detection range: 0.5–100 ng/mL, sensitivity: 0.28 ng/mL), p-mTOR (Cat. No. E3497Ra, BT LAB; detection range: 18.75–1200ng/L, sensitivity: 10.6 ng/L), total mTOR (Cat. No. E2096Ra, BT LAB; detection range: 5–1500 ng/L, sensitivity: 2.41 ng/L), and NF-κB (Cat. No. RE3044R, Reed Biotech Ltd., Wuhan, China; detection range: 78.13–50000 pg/mL, sensitivity: 46.88 pg/mL) were quantified in tissue supernatants. To ensure optimal measurement within the linear range of the calibration curves, serum and tissue supernatant samples were appropriately diluted with the assay diluent provided in the respective kits. All samples, standards, and controls were analyzed in duplicate. Intra-assay and inter-assay coefficients of variation (CV) for all kits were <8% and <10%, respectively. Furthermore, laboratory personnel conducting the biochemical assays were blinded to group allocations until all statistical analyses were initiated.

### 2.7. Statistical Analysis

Statistical analyses were performed using a web-based statistical software developed by Arslan et al. [43]. The software is freely available at https://biostatapps.inonu.edu.tr/ (İnönü University, Malatya, Türkiye). A power analysis was performed to determine the required sample size. Based on the right ventricular systolic pressure data from studies in the literature [33,34], in order to determine a significant difference between groups, it was concluded that the minimum sample size required to find a significant difference using the Independent Samples One-Way Analysis of Variance test was 10 for each group, with a Type I error of 0.05 (alpha), test power (1-beta) of 0.8, and effect size of 0.52. Considering the high mortality rate of the pulmonary arterial hypertension model, the number of animals was determined as 10 for the control group; 12 for the 4 groups in which PAH was induced; and a total of 58.

The data obtained from the experiments were statistically analyzed. The normality of data distribution was assessed using the Shapiro–Wilk test; detailed *W* statistics and the corresponding *p* values for each parameter and group are provided in Tables S1–S5 in the Supplementary Data. To facilitate assessment of variability and potential outliers, individual animal-level raw data underlying all figures are additionally provided in Supplementary Tables S6–S10. For comparisons among multiple groups, one-way analysis of variance (ANOVA) was applied for normally distributed data, followed by Tukey’s or Tamhane’s T2 post hoc tests depending on the homogeneity of variances. For data that did not meet the assumption of normality, the Kruskal–Wallis H test was used, followed by Conover’s post hoc test with Holm correction for pairwise comparisons when the omnibus test was significant. All pairwise comparisons reported in the figures (versus control, MCT, α-LA, and, where relevant, AMB) were prespecified rather than exploratory, and multiplicity within each family of comparisons was controlled by the post hoc procedure used (Tukey’s, Tamhane’s T2, or Conover–Holm, as appropriate). For the echocardiographic parameters (Section 3.1; Table 5), RVSP and HR (Section 3.2; Table 6), the Fulton index (Section 3.3; Table 7), the histopathological and immunohistochemical parameters (Section 3.4), and the ELISA parameters (Section 3.5; Table 9), effect sizes (rank-biserial correlation, r) and 95% confidence intervals (Hodges–Lehmann estimator of the median difference) were additionally calculated for all pairwise comparisons using the Mann–Whitney U/Wilcoxon rank-sum framework to characterize the magnitude and precision of the observed differences (Table 5, Table 6, Table 7, Table 8 and Table 9); these effect-size estimates were derived independently of, and are not corrected for multiplicity by, the Conover–Holm procedure used for hypothesis testing, so the exact *p* value accompanying each effect size may differ slightly from the corresponding Conover–Holm post hoc *p* value reported in the text and figures, although the direction and overall pattern of significance are consistent between the two approaches. A *p* value < 0.05 was considered statistically significant. Data are presented as mean ± standard deviation (SD) for normally distributed variables and as median (interquartile range) for non-normally distributed variables. Sample sizes (n) utilized for each statistical test are explicitly reported in the text, table legends, and figure captions per ARRIVE guidelines. Graphs were generated using GraphPad Prism version 8.0.2 (GraphPad Software, San Diego, CA, USA). In all figures, statistically significant pairwise differences between groups (*p* < 0.05) are indicated using superscript letters (a, b, c, d), each explicitly defined in the corresponding figure legend (e.g., a: vs. control group; b: vs. MCT group); the absence of a letter for a given comparison indicates no statistically significant difference.

## 3. Results

### 3.1. Echocardiography

**Figure 3:**
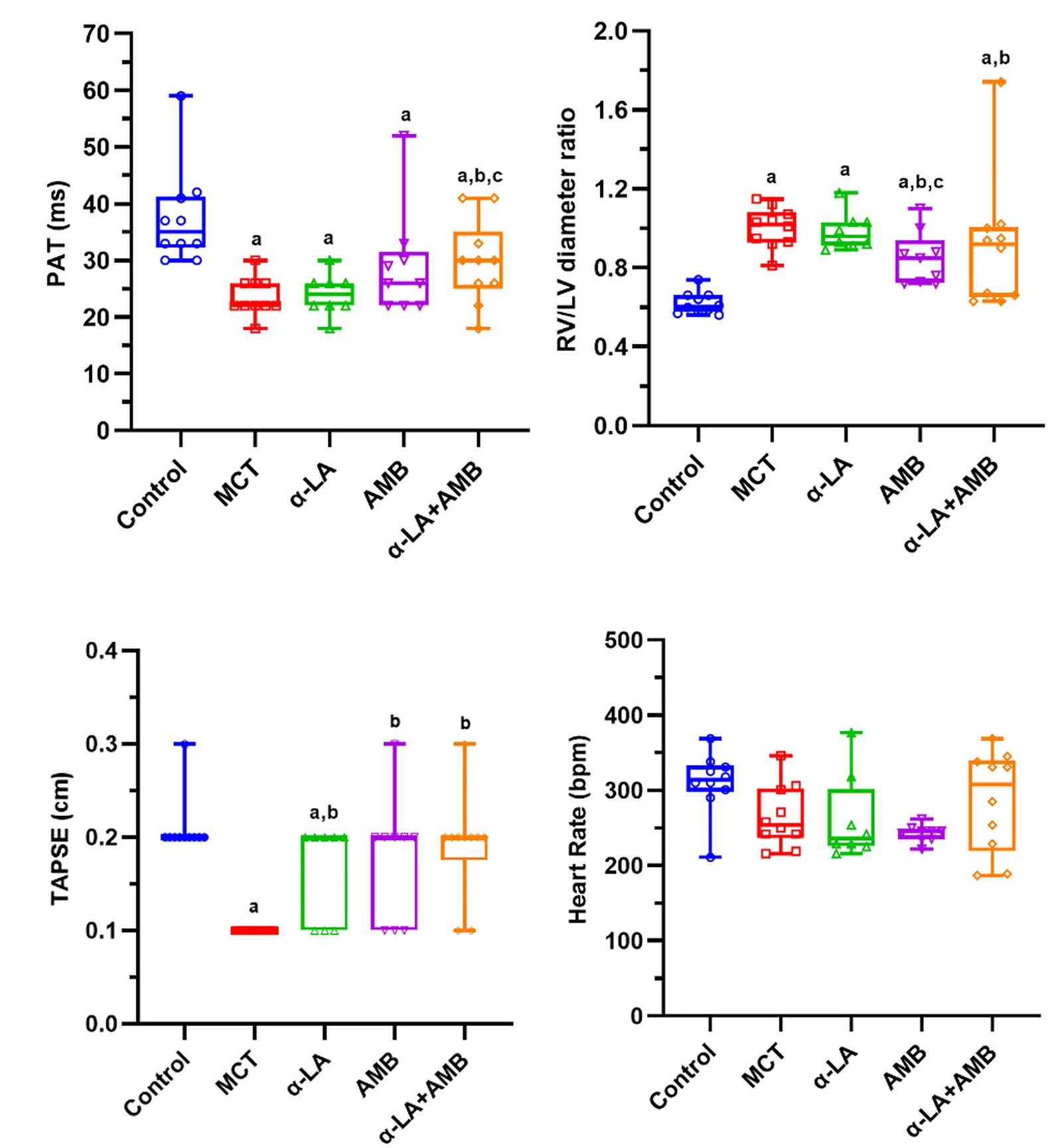
Intergroup comparison of echocardiographic parameters. Sample sizes: Control (*n* = 10), MCT (*n* = 10), α-LA (*n* = 8), AMB (*n* = 9), α-LA + AMB (*n* = 10). MCT: Monocrotaline-treated group, α-LA: Alpha-lipoic acid-treated group, AMB: ambrisentan-treated group, α-LA + AMB: Alpha-lipoic acid-and-ambrisentan-treated group, PAT: pulmonary acceleration time, RV: right ventricle, LV: left ventricle, TAPSE: tricuspid annular plane systolic excursion, ms: millisecond, cm: centimeter. Kruskal–Wallis H test, followed by Conover’s post hoc test with Holm correction, was used in the statistical analyses. a: Difference vs. control group (*p* < 0.05), b: Difference vs. MCT group (*p* < 0.05), c: Difference vs. α-LA group (*p* < 0.05).

**Figure 4:**
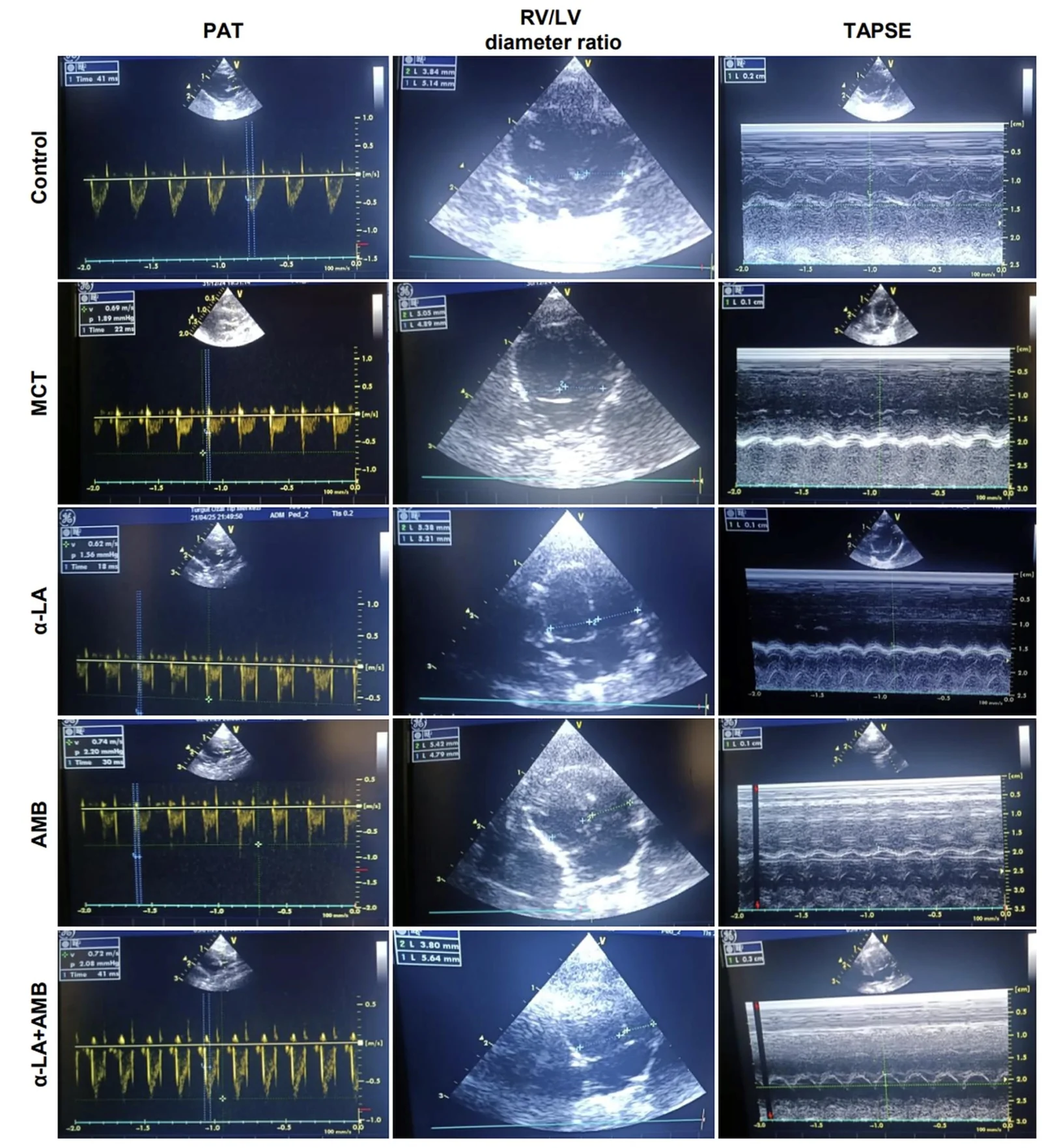
Representative echocardiographic images illustrating the measurement of pulmonary artery acceleration time (PAT, left column), right ventricle-to-left ventricle diameter ratio (RV/LV, middle column), and tricuspid annular plane systolic excursion (TAPSE, right column) in the Control, MCT, α-LA, AMB, and α-LA + AMB groups (rows, top to bottom). PAT was measured from the parasternal short-axis view using pulsed-wave Doppler within the right ventricular outflow tract (RVOT); RV/LV diameter ratio was measured in the apical four-chamber view; TAPSE was measured in the RV-focused apical four-chamber view using M-mode imaging along the lateral tricuspid annulus. A mid-systolic notch in the pulmonary flow velocity envelope, indicative of increased pulmonary vascular resistance, was observed in the MCT, α-LA, and AMB groups but was absent in the Control and α-LA + AMB groups. Representative values were as follows—Control: PAT 41 ms, RV/LV 0.75 (LV 5.14 mm, RV 3.84 mm), TAPSE 0.2 cm; MCT: PAT 22 ms, RV/LV 1.03 (LV 4.89 mm, RV 5.05 mm), TAPSE 0.1 cm; α-LA: PAT 18 ms, RV/LV 1.03 (LV 5.21 mm, RV 5.38 mm), TAPSE 0.1 cm; AMB: PAT 30 ms, RV/LV 1.13 (LV 4.79 mm, RV 5.42 mm), TAPSE 0.1 cm; α-LA + AMB: PAT 41 ms, RV/LV 0.67 (LV 5.64 mm, RV 3.80 mm), TAPSE 0.3 cm. MCT: Monocrotaline-treated group; α-LA: Alpha-lipoic acid-treated group; AMB: ambrisentan-treated group; α-LA + AMB: Alpha-lipoic acid-and ambrisentan-combination-treated group; PAT: pulmonary acceleration time; RV: right ventricle; LV: left ventricle; TAPSE: tricuspid annular plane systolic excursion; RVOT: Right ventricle outflow tract; M-mode: Motion mode; 1 L and 2 L: LV and RV diameters, respectively; Time: PAT; V: probe orientation marker; v: velocity; p: pressure; ms: millisecond; m/s: Meter per second; mmHg: Millimeter of mercury; cm: centimeter; mm: Millimeter; mm/s: Millimeter per second.

The results of the transthoracic echocardiogram are presented in Figure 3 (analyzed sample size: Control *n* = 10, MCT *n* = 10, α-LA *n* = 8, AMB *n* = 9, α-LA + AMB *n* = 10). According to these results, compared to the control group, PAT and TAPSE were significantly reduced in the MCT group, while the RV/LV diameter ratio was significantly increased. In the α-LA group, TAPSE was measured higher than in the MCT group, although it remained significantly lower than in the control group.The AMB group stood out as the group with the most improvement in the RV/LV diameter ratio. In addition, TAPSE was also measured higher compared to the MCT group. In the group receiving the combination of α-LA and AMB, all three echocardiography parameters were found to have improved significantly compared to the MCT group. Direct comparison of the α-LA + AMB group against each monotherapy showed a significantly higher PAT than α-LA alone (*p* < 0.05), but no significant difference from AMB; RV/LV diameter ratio and TAPSE did not differ significantly between α-LA + AMB and either monotherapy. Echocardiographic images of rats in the MCT group and α-LA + AMB are presented in Figure 4.

Heart rate recorded during echocardiographic acquisition is reported and compared between groups in Table 5 below.

### 3.2. RVSP

**Figure 5:**
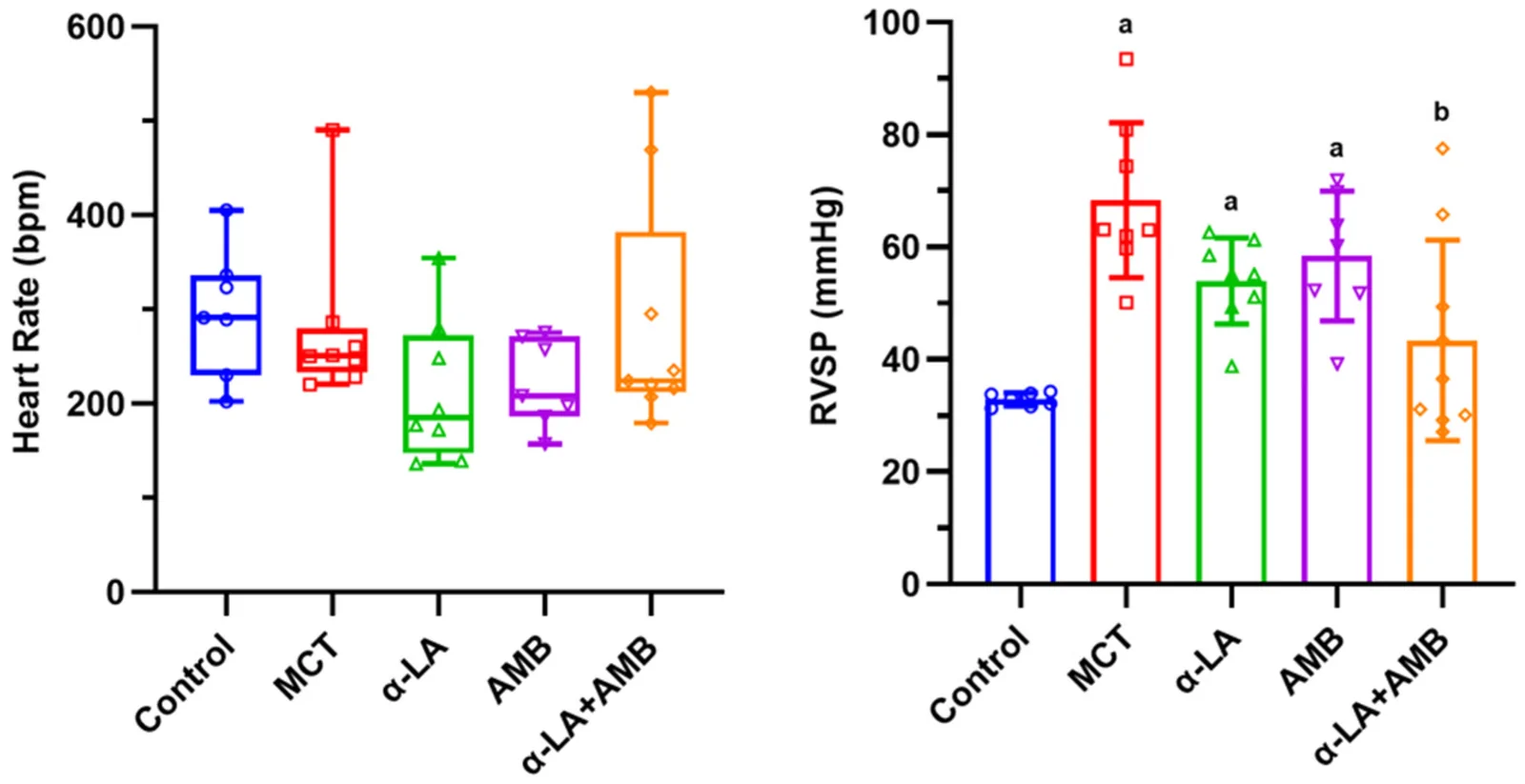
Comparison of RVSP values across study groups. Sample sizes: Control (*n* = 7), MCT (*n* = 8), α-LA (*n* = 8), AMB (*n* = 7), α-LA + AMB (*n* = 9). a: Difference vs. control group (*p* < 0.05), b: Difference vs. MCT group (*p* < 0.05). One-Way ANOVA test, followed by Tukey’s HSD post hoc test or Kruskal–Wallis H test, followed by Conover’s post hoc test with Holm correction was used in the statistical analyses.

**Figure 6:**
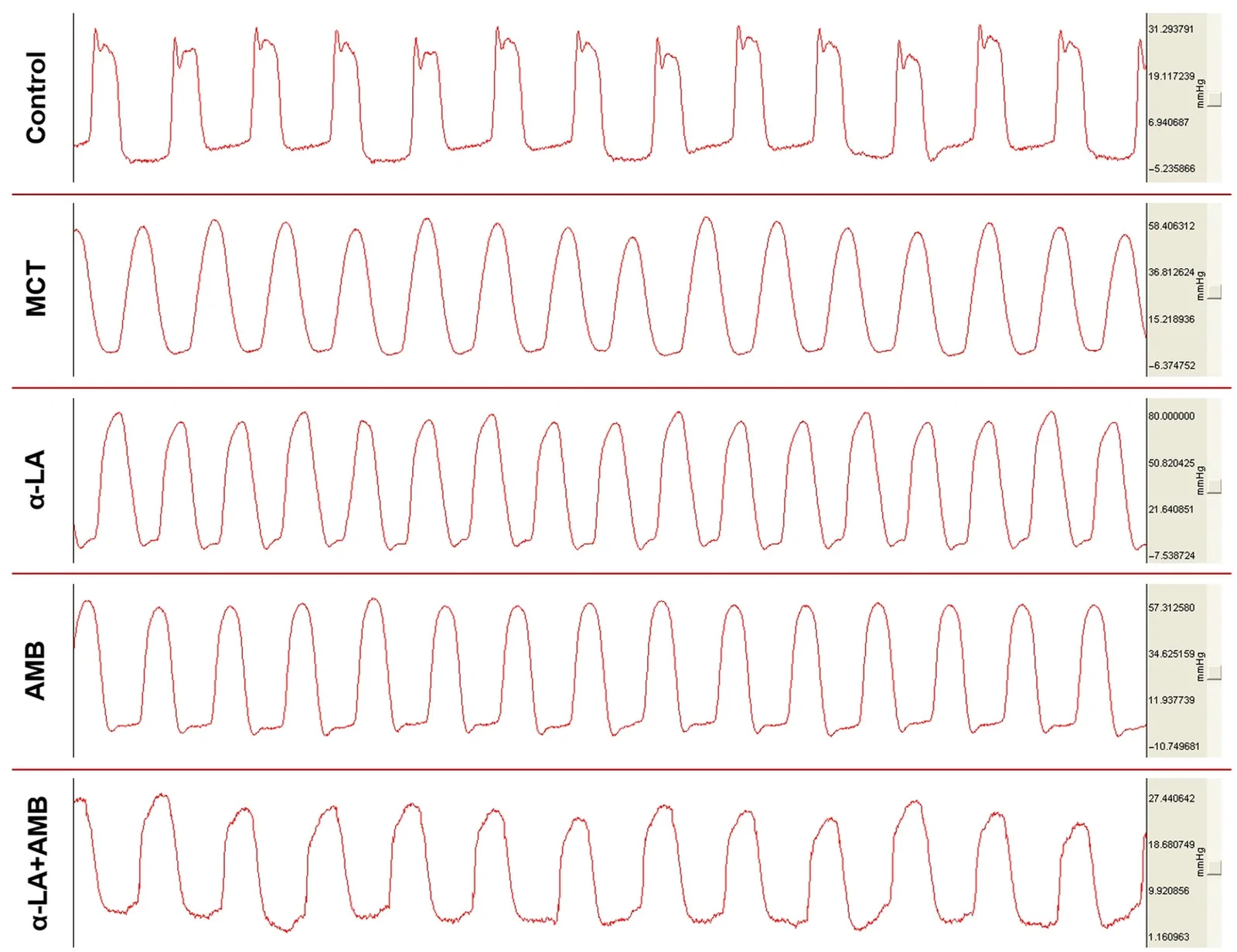
Representative right ventricular pressure waveforms, recorded via jugular venous catheterization, are shown for each experimental group (Control, MCT, α-LA, AMB, and α-LA + AMB), illustrating the recordings underlying the quantitative RVSP data.

Hemodynamic evaluations of RVSP were conducted in valid recordings (Control *n* = 7, MCT *n* = 8, α-LA *n* = 8, AMB *n* = 7, α-LA + AMB *n* = 9). Compared to the control group, statistically significant increases in RVSP measurements were observed in the MCT, α-LA, and AMB groups. No significant change was observed between the α-LA + AMB treatment group and the control group, and the measurements were close to each other. In addition, a significant decrease in RVSP was observed in the α-LA + AMB group compared to the MCT group. There were no significant differences in RVSP measurements between the MCT, α-LA, and AMB groups. Direct comparisons of the α-LA + AMB group against each monotherapy (α-LA and AMB) were also performed and did not reach statistical significance (*p* ≥ 0.05) (Figure 5). Representative RV pressure waveforms recorded via jugular venous catheterization in each group are shown in Figure 6.

Heart rate recorded during invasive RVSP measurement is also reported and compared between groups in Table 6 below.

### 3.3. Fulton Index

**Figure 7:**
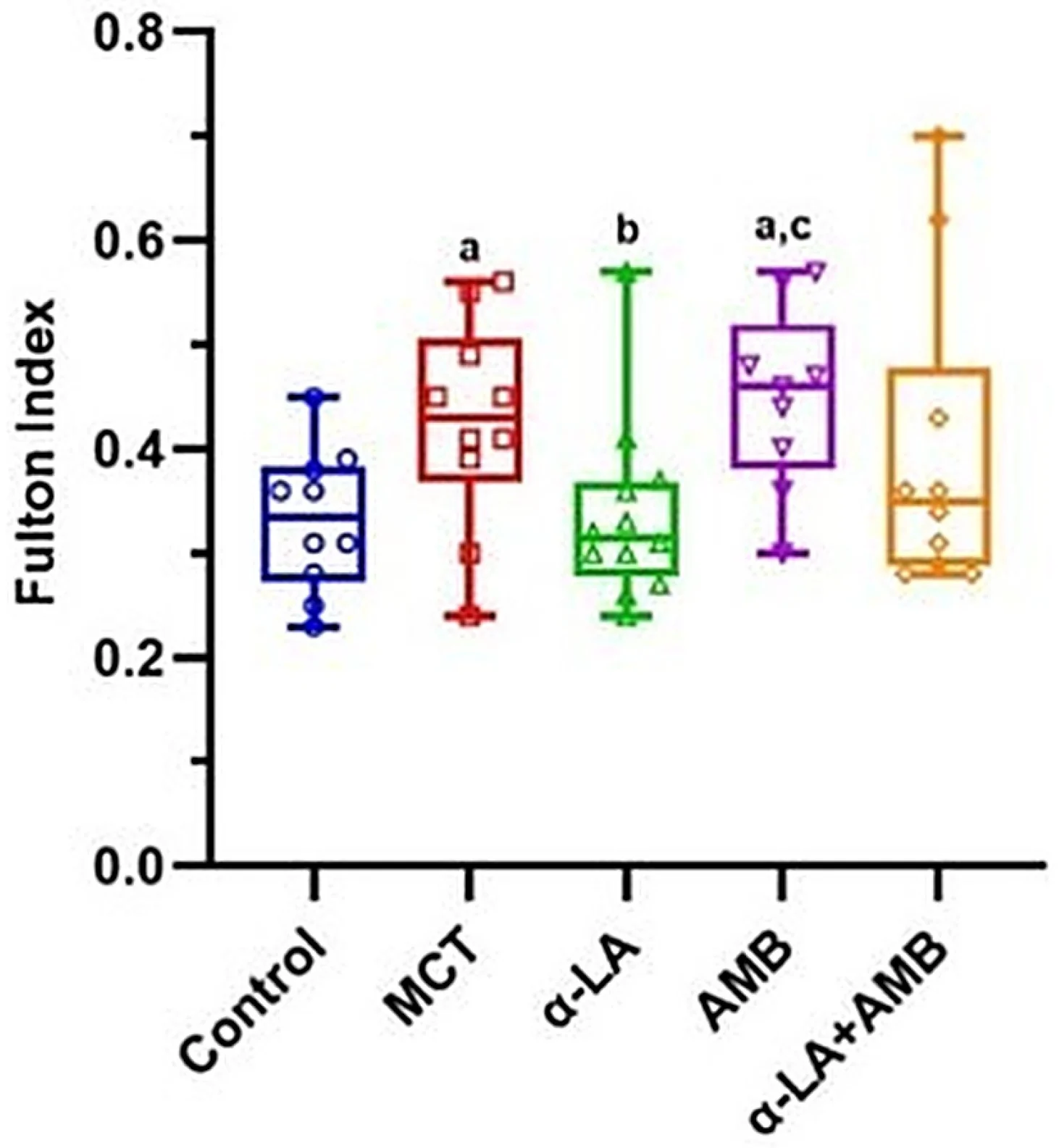
Assessment of right ventricular hypertrophy using the Fulton index in experimental groups. Sample sizes: Control (*n* = 10), MCT (*n* = 10), α-LA (*n* = 12), AMB (*n* = 9), α-LA + AMB (*n* = 10). The Kruskal–Wallis H test, followed by Conover’s post hoc test with Holm correction, was used for statistical analyses. a: Difference vs. control group (*p* < 0.05), b: Difference vs. MCT group (*p* < 0.05), c: Difference vs. α-LA group (*p* < 0.05).

The Fulton index (RV/(LV + IVS)) (Control *n* = 10, MCT *n* = 10, α-LA *n* = 12, AMB *n* = 9, α-LA + AMB *n* = 10) was higher in the MCT group compared with the control group (*p* < 0.05). A reduction was observed in the α-LA and α-LA + AMB groups compared with the MCT group, particularly in the α-LA group. The decrease in the α-LA group reached statistical significance (Figure 7). In contrast, the Fulton index in the AMB group did not differ significantly from the MCT group (*p* = 0.573) and remained significantly higher than in the control (*p* = 0.012) and α-LA (*p* = 0.009) groups, and the reduction observed in the α-LA+AMB group relative to MCT did not reach statistical significance (*p* = 0.243). Direct comparison showed that α-LA + AMB did not differ significantly from either α-LA or AMB monotherapy for the Fulton index.

### 3.4. Histopathology

**Figure 8:**
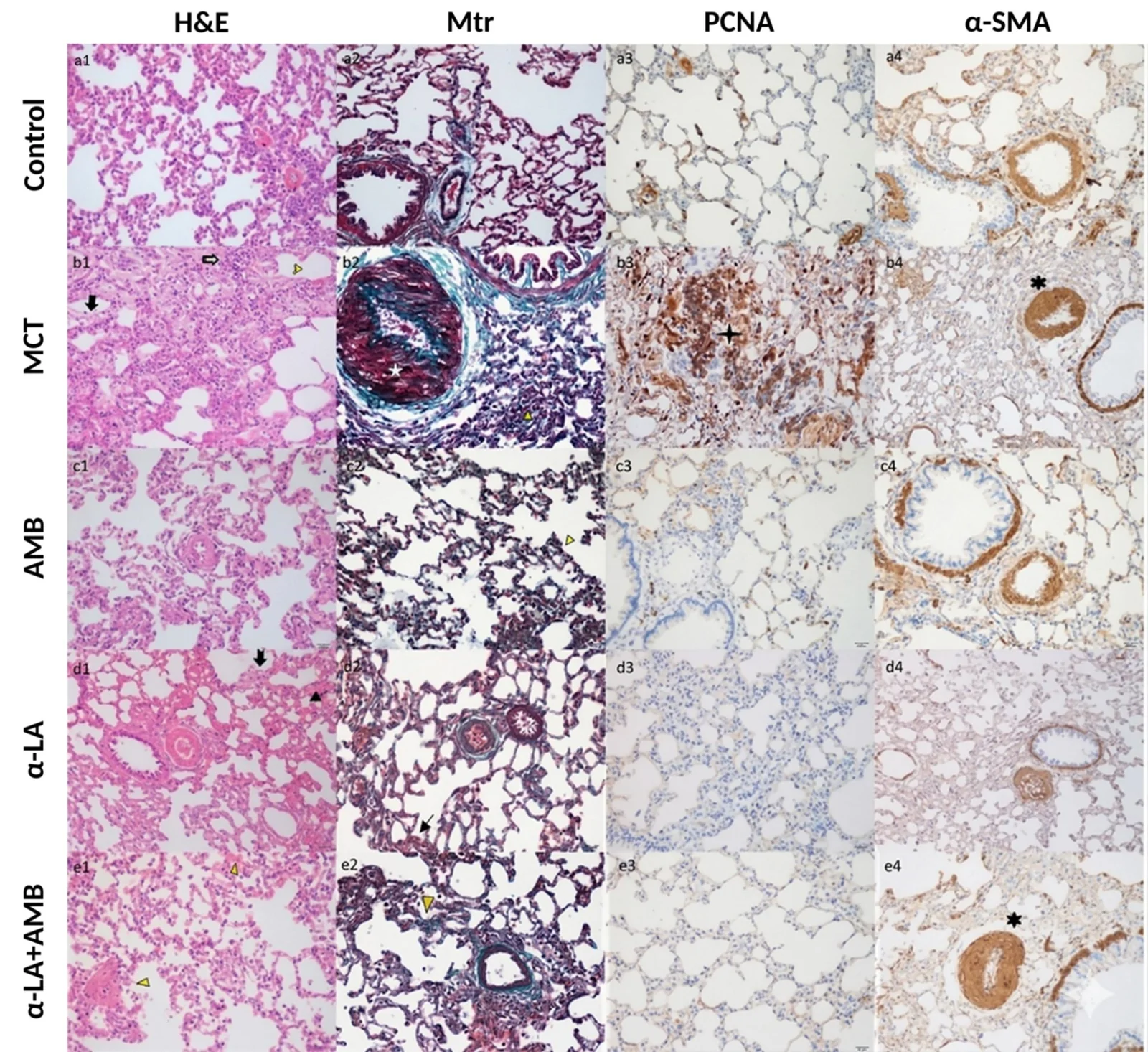
(**a1**,**a2**) Normal lung histology, H&E, Masson’s trichrome (Mtr), ×20. (**a3**) Low or negative staining intensity of the proliferation marker, PCNA, ×20. (**a4**) Moderate staining intensity of the smooth muscle biomarker, α-SMA, ×20. (**b1**,**b2**) Findings of pulmonary hypertension, including neutrophil leukocyte and mononuclear cell infiltration (striped arrow), activated alveolar macrophages (notched arrow), interstitial thickening and fibrosis (arrowhead), hyperplastic tunica media, and vascular wall thickening (asterisk), H&E, Mtr, ×20. (**b3**) Alveolar cells forming multiple layers within the alveolar septa, demonstrating proliferation in areas of interstitial thickening (four-pointed star), PCNA, ×20. (**b4**) Smooth muscle cell hypertrophy with marked thickening of the medial layer; intense staining of the smooth muscle marker (six-pointed star), α-SMA, ×10. (**c1**,**c2**) Mild degenerative changes with overall normal histology; partially increased collagen deposition in the alveolar septa (arrowhead), H&E, Mtr, ×20. (**c3**) Mild or negative staining of the proliferation marker, PCNA, ×20. (**c4**): Moderate staining intensity of the smooth muscle marker in the tunica media, α-SMA, ×20. (**d1**,**d2**) Focal, low-grade interstitial thickening (thin arrow) with sparse alveolar macrophages (notched arrow), H&E, Mtr, ×20. (**d3**) Negative staining for the proliferation marker, PCNA, ×20. (**d4**) Moderate staining intensity of the smooth muscle marker in the tunica media, α-SMA, ×20. (**e1**,**e2**) Focal, low-grade interstitial thickening with a moderate increase in collagen deposition (arrowhead), H&E, Mtr, ×20. (**e3**) Negative staining for the proliferation marker, PCNA, ×20. (**e4**) Marked thickening of the medial layer with intense staining of the smooth muscle marker (six-pointed star), α-SMA, ×20. Rows correspond to the experimental groups: (a) Control, (b) MCT, (c) AMB, (d) α-LA, and (e) α-LA+AMB.

**Figure 9:**
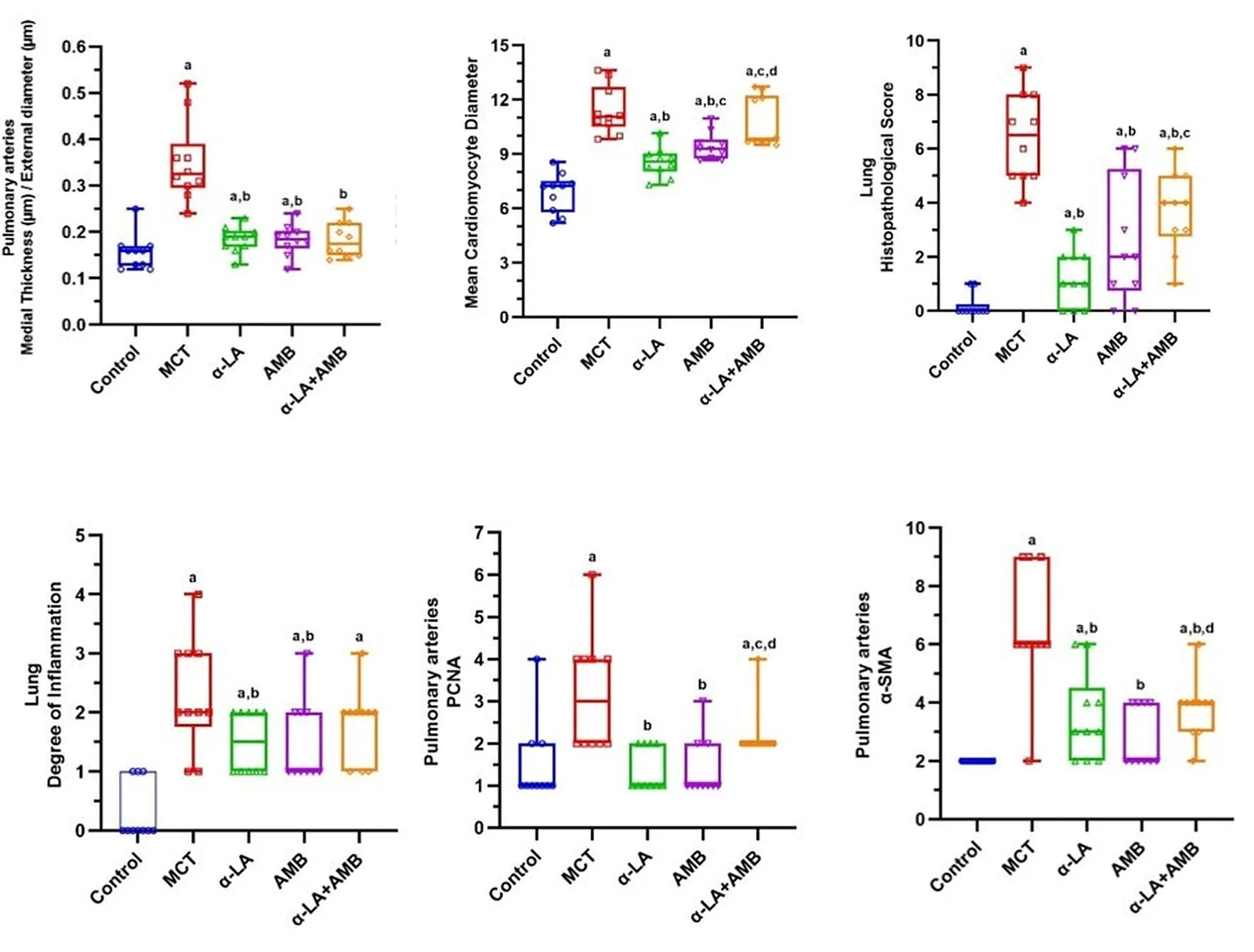
The impact of α-LA, AMB, and α-LA + AMB treatments on histopathological parameters. Sample sizes: *n* = 10 per group. PCNA: Proliferating cell nuclear antigen; α-SMA: Alpha–smooth muscle actin. The Kruskal–Wallis H test, followed by Conover’s post hoc test with Holm correction, was used for statistical analyses. a: Difference vs. control group (*p* < 0.05), b: Difference vs. MCT group (*p* < 0.05), c: Difference vs. α-LA group (*p* < 0.05), d: Difference vs. AMB group (*p* < 0.05).

Lung tissues of the control group exhibited normal histological appearance (Figure 8(a1)). All histopathological and immunohistochemical parameters were evaluated in *n* = 10 animals per group. Detailed data on medial thickness (µm)/external diameter (µm) (PAs: pulmonary arteries), mean cardiomyocyte diameter, histopathological score (lung), degree of inflammation (lung), PCNA (PAs), and α-SMA (PAs) parameters in the control group are presented in Figure 9. In Masson’s trichrome–stained sections, no evidence of interstitial fibrosis, medial hypertrophy, or inflammation was observed (Figure 8(a2)). The presence of foamy activated alveolar macrophages was detected in one tissue sample. Following IHC staining, PCNA expression was evaluated as weak or negative (Figure 8(a3)), while α-SMA showed moderate cytoplasmic staining intensity (Figure 8(a4)). Compared with the control group, the MCT group demonstrated a significant increase in medial thickness (µm)/external diameter (µm) (PAs), mean cardiomyocyte diameter, histopathological score (lung), degree of inflammation (lung), PCNA (PAs), and α-SMA (PAs) parameters (*p* < 0.05) (Figure 9). Histopathological analysis of the MCT group revealed a predominantly hyperplastic appearance of the tunica media layer in pulmonary and respiratory bronchiolar arteries, as demonstrated by α-SMA staining (Figure 8(b4)). In some arteries, collagen-rich fibrosis was notable in the adventitial layer. Tissue samples from this group frequently showed inflammatory findings accompanied by neutrophil infiltration and activated foamy alveolar macrophages (Figure 8(b1,b2)). Although focal interstitial thickening was observed in some tissues of the MCT group, plexogenic arteriopathy was not detected in any sample. A marked increase in PCNA and α-SMA staining intensity was observed (Figure 8(b3,b4) and Figure 9). Histopathological alterations were significantly milder in the α-LA and AMB monotherapy groups compared with the MCT group, with significant improvement observed in all histological parameters in these groups (*p* < 0.05) (Figure 8 and Figure 9). In the AMB group, the lowest degree of inflammatory findings was observed numerically among the treatment groups, although the difference from the α-LA group was not statistically significant (*p* = 0.878), and respiratory bronchioles and alveolar structures exhibited a normal histological appearance overall (Figure 8(c1,c2)). In the α-LA group, in addition to sparse medial thickening and inflammatory findings, focal interstitial thickening observed in two tissues was notable on Masson’s trichrome staining (Figure 8(d2)); although the overall histological appearance of this group approached that of the control group, α-LA-treated rats still differed significantly from controls in five of the six quantitative parameters (all except PCNA; Figure 9 and Table 8). α-LA monotherapy resulted in the lowest cardiomyocyte diameter and histopathological score among the treatment groups; cardiomyocyte diameter was significantly lower than in the AMB group (*p* = 0.015), whereas the difference in histopathological score versus AMB was borderline and not statistically significant (*p* = 0.050).In the AMB+α-LA group, the tunica media layer was evaluated as hyperplastic compared with the control, AMB, and α-LA groups; fibrosis in the adventitial layer was noted in some arteries, along with focal neutrophil infiltration and activated foamy alveolar macrophages. In the combination therapy group, significant improvements were determined in medial thickness/external diameter, histopathological score, and α-SMA expression, whereas mean cardiomyocyte diameter (*p* = 0.122), degree of inflammation (*p* = 0.224), and PCNA expression (*p* = 0.178) did not differ significantly from the MCT group (Table 8).

Direct comparison of the α-LA + AMB group against each monotherapy showed the following pattern for the histopathological and immunohistochemical parameters. Medial thickness/external diameter and degree of inflammation did not differ significantly between α-LA + AMB and either α-LA or AMB. Mean cardiomyocyte diameter and PCNA were significantly higher (i.e., less improved) in the α-LA + AMB group than in both the α-LA and AMB groups. Histopathological score was significantly higher in the α-LA+AMB group than in the α-LA group but did not differ significantly from the AMB group, whereas α-SMA expression did not differ significantly from the α-LA group but was significantly higher than in the AMB group. Taken together, for several proliferation- and remodeling-related parameters, the combination showed an attenuated benefit relative to α-LA monotherapy rather than an additive or superior effect.

### 3.5. ELISA

**Figure 10:**
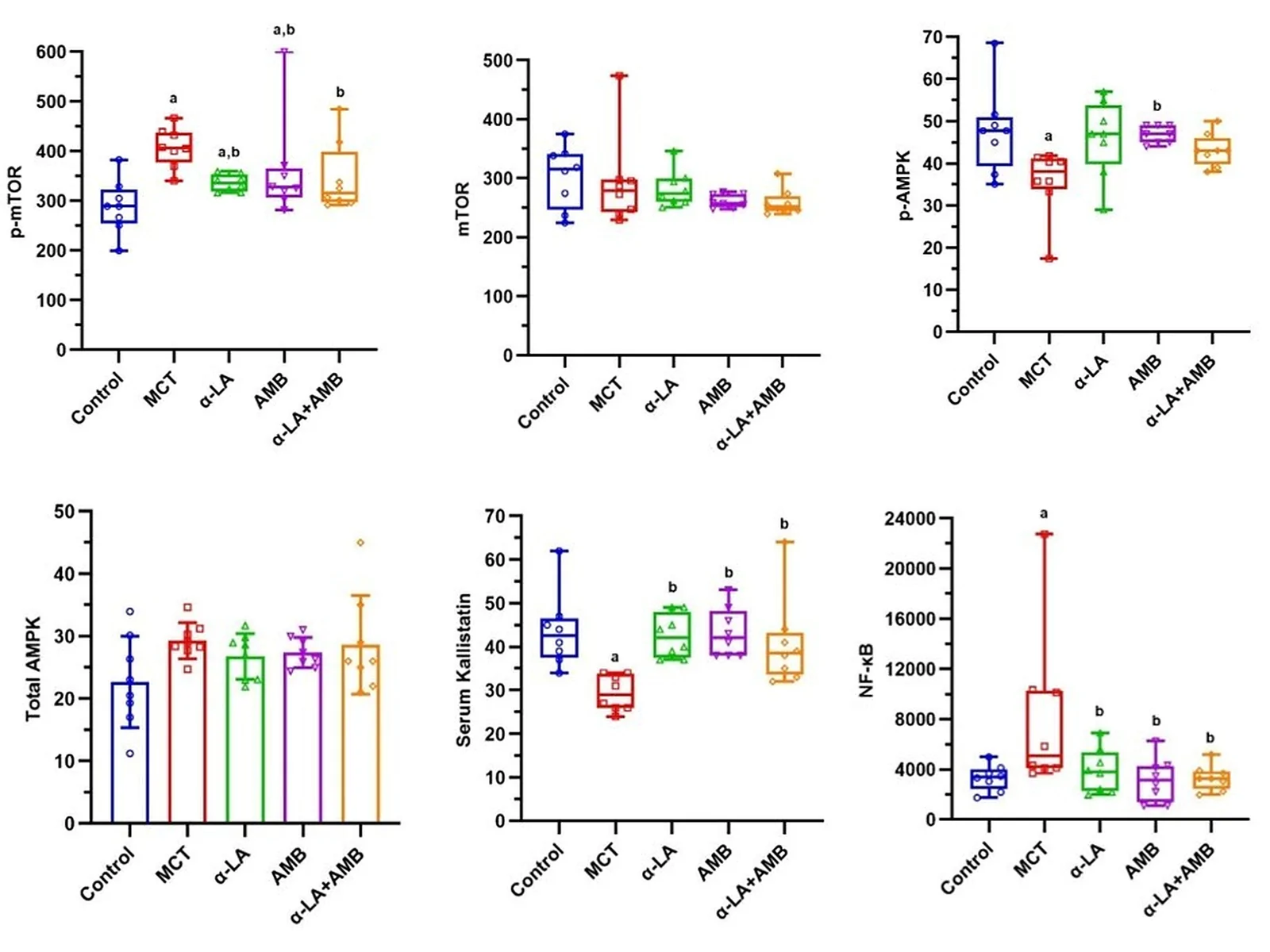
Comparison of p-mTOR, mTOR, p-AMPK, total AMPK, serum kallistatin, and NF-κB biochemical parameters among the groups. Sample sizes: *n* = 8 per group. p-mTOR: phosphorylated-mechanistic target of rapamycin, mTOR: Mechanistic target of rapamycin, p-AMPK: phosphorylated-AMP-activated protein kinase, Total AMPK: Total AMP-activated protein kinase, NF-κB: Nuclear factor kappa-B. The one-way analysis of variance (ANOVA) test, followed by Tukey’s HSD post hoc test, was used for normally distributed data; the Kruskal–Wallis H test, followed by Conover’s post hoc test with Holm correction, was used for data that did not meet the assumption of normality. a: Difference vs. control group (*p* < 0.05), b: Difference vs. MCT group (*p* < 0.05).

Our ELISA results, quantified in lung tissue homogenates (kallistatin in serum), are presented in detail in Figure 10 (analyzed sample size: *n* = 8 per group for all target proteins). According to the data, the MCT group showed a significant increase in p-mTOR and NF-κB levels and a significant decrease in p-AMPK and serum kallistatin levels compared with the control group (*p* < 0.05). In all treatment groups, a significant decrease in p-mTOR and NF-κB levels and a significant increase in serum kallistatin levels were observed compared with the MCT group (*p* < 0.05). p-AMPK levels were higher in all treatment groups than in the MCT group; however, this increase was statistically significant only in the AMB group (*p* = 0.035) and did not reach significance in the α-LA (*p* = 0.062) or α-LA+AMB (*p* = 0.267) groups.No significant differences were observed among the groups with respect to mTOR and total AMPK levels. Direct comparisons of the α-LA + AMB group against each monotherapy did not reach statistical significance for any of the measured biochemical parameters (p-mTOR, mTOR, p-AMPK, total AMPK, kallistatin, NF-κB).

Absolute concentrations of p-AMPK and p-mTOR, rather than phosphorylation ratios (p-AMPK/total AMPK, p-mTOR/total mTOR), were used as the primary biochemical readouts in this analysis. Because total AMPK and total mTOR did not differ significantly among groups, the direction of the absolute phosphorylated-protein findings is expected to parallel the corresponding ratios; nevertheless, ratio-based analyses were not performed, and this should be considered when interpreting the magnitude of pathway activation.

## 4. Discussion

This study investigated the therapeutic potential of α-LA alone and in combination with AMB in a rat model of MCT-induced PAH. For this purpose, treatment groups received α-LA 100 mg/kg/day s.c. and/or AMB 0.2 mg/kg/day p.o. for 14 days. The study was enriched by RVSP measurement, echocardiographic and histopathological evaluations, as well as ELISA examinations. To our knowledge, this is among the first studies to investigate the effects of α-LA in an experimental pulmonary arterial hypertension model; this claim is based on our literature review at the time of writing and cannot exclude unindexed or very recent studies.

In this study, combination therapy significantly reduced MCT-induced RVSP increase, and, unlike either monotherapy, the α-LA + AMB group did not differ significantly from control, suggesting a trend toward more complete normalization; however, direct comparison of α-LA + AMB versus α-LA and versus AMB did not reach statistical significance for RVSP, so this apparent advantage is not formally confirmed. RVSP is a principal hemodynamic endpoint in experimental PH models [33,34]. α-LA’s known antihypertensive effects may partly explain the modest benefit observed. For example, Dudek et al. reported that a single dose of 50 mg/kg i.p. α-LA exerts a hypotensive effect via ATP-dependent potassium channels or hydrogen sulfide in rats [44]. Moreover, Vasdev et al. reported that an α-LA diet (500 mg/kg feed for 9 weeks) lowers systolic blood pressure and attenuates renal vascular hyperplasia in spontaneously hypertensive rats [45].

Echocardiographically, low PAT indicates PH [1]. Combination therapy significantly attenuated the MCT-induced PAT reduction; direct comparison showed that α-LA + AMB achieved significantly higher PAT than α-LA alone, although it did not differ significantly from AMB monotherapy. A study on children with PH demonstrated that increased RV/LV DIAM correlates with pulmonary artery pressures and RV remodeling, including dilation [46]. Similarly, in our study, elevated RV/LV DIAM is a finding that supports RV remodeling and, therefore, PAH. In this study, AMB and combination therapy significantly reduced MCT-induced RV/LV DIAM increase, whereas α-LA monotherapy did not significantly change it (*p* = 0.548). Moreover, a reduced TAPSE is a validated marker of RV dysfunction and is linked to PH [1, 47]. In our study, all treatments significantly attenuated MCT-induced TAPSE decrease. Taken together, the echocardiographic findings indicate that combination therapy improved RV function relative to MCT to an extent broadly similar to monotherapy, with a significant advantage over α-LA alone observed only for PAT; direct comparisons did not demonstrate superiority of the combination over either monotherapy for RV/LV diameter ratio or TAPSE. To our knowledge, the effects of α-LA on RV function were unknown, as previous studies have primarily focused on LV function, showing improved systolic and diastolic dysfunction in type 1 diabetes [48].

α-LA monotherapy significantly reduced the PAH-induced increase in the Fulton index, indicating amelioration of RVH, supported by echocardiographic and histopathological data. It also attenuated the elevated mean cardiomyocyte diameter, suggesting anti-hypertrophic effects, although it did not significantly reduce the RV/LV diameter ratio (*p* = 0.548). In contrast, neither AMB monotherapy nor combination therapy significantly reduced the Fulton index relative to MCT (*p* = 0.573 and *p* = 0.243, respectively), and the Fulton index in the AMB group remained significantly higher than in the control and α-LA groups. Thus, despite improvements in RVSP and/or echocardiographic indices with AMB-containing regimens, a significant regression of RV hypertrophy was observed only with α-LA monotherapy; the relatively short 14-day treatment period may have been insufficient for regression of established RV hypertrophy. Previous studies report similar findings: α-LA reduces phenylephrine-induced cardiomyocyte hypertrophy, mitigates hemodynamic and hypertrophic responses after coronary artery ligation, likely via inhibition of C/EBPβ [49], and attenuates vascular smooth muscle hyperplasia and arterial wall thickening in the kidney [45].

MCT induced pulmonary arterial remodeling, evidenced by increased medial thickness/external diameter and elevated PCNA and α-SMA, indicating PASMC proliferation and hypertrophy. Both α-LA and AMB monotherapies significantly improved these changes, showing antiproliferative, anti-hypertrophic, and anti-remodeling effects. Combination therapy significantly reduced medial thickness/external diameter and histopathological score relative to MCT; however, direct comparison showed no significant difference from either monotherapy for medial thickness, whereas histopathological score and cardiomyocyte diameter in the combination group were significantly higher (i.e., less improved) than in the α-LA group (though not significantly different from AMB for histopathological score), and α-SMA expression was significantly higher than in the AMB group but did not differ significantly from the α-LA group.Notably, PCNA expression in the combination group was significantly higher than in both the α-LA and AMB monotherapy groups, indicating an attenuated antiproliferative effect when the two agents were combined. MCT-induced pulmonary inflammation was significantly reduced by α-LA and AMB monotherapies, with no significant difference between the two monotherapies (*p* = 0.878); the degree of inflammation in the combination group did not differ significantly from either monotherapy and was not significantly reduced relative to MCT (*p* = 0.224). Interestingly, despite a comparable or occasionally greater hemodynamic benefit (e.g., PAT versus α-LA alone), the addition of AMB did not enhance—and for several proliferation- and remodeling-related markers (cardiomyocyte diameter, PCNA, histopathological score) significantly reduced—the antiproliferative and anti-remodeling benefit of α-LA monotherapy. In some parameters, these effects appeared attenuated after AMB addition, which may reflect a non-additive pharmacodynamic interaction between the two agents. Although α-LA and AMB have different primary targets, their downstream effects may converge on common pathways involved in inflammation and pulmonary vascular smooth muscle cell proliferation. However, this explanation remains hypothetical, as drug–drug interactions were not directly investigated. Further studies are needed to clarify the interaction between α-LA and AMB.

Increased proliferation in pulmonary arteries is a key factor in PAH pathogenesis, with hPASMC proliferation and migration driving vascular remodeling and elevated PVR [50]. The AMPK/mTOR pathway plays a critical role in regulating cell proliferation, as AMPK negatively regulates mTOR [51]. AMPK controls cell proliferation and vascular tone, inhibiting smooth muscle contraction via mechanisms such as suppression of myosin light chain kinase, RhoA/ROCK, protein kinase C, or intracellular Ca^2+^ [25, 52, 53]. AMPK is also important in PH development: endothelial AMPK knockout in mice increased hypoxia-induced PH, growth factor levels (PDGF-BB, FGF-2), and PASMC proliferation, while human PAH patients show decreased endothelial AMPK activity in distal pulmonary arteries [52]. Consistent with these findings, our study shows reduced lung AMPK activity in PAH. Similarly, Abdelazeem et al. found that lung AMPK activity was diminished in an MCT-induced PAH model. They also showed that the AMPK activator metformin significantly lowered mPAP, total PVR, and the Fulton index. In their research, metformin reduced mPAP, pulmonary vascular remodeling, RV hypertrophy, and fibrosis. Additionally, metformin decreased medial wall thickness in distal pulmonary arteries and lowered the number of PCNA-positive cells in these arteries, thus mitigating pulmonary vascular remodeling and PASMC proliferation. It also reduced collagen buildup in the RV. They also reported an inverse correlation between mPAP and AMPK phosphorylation, indicating that higher AMPK activity is associated with lower mPAP [53].

AMPK regulates mammalian target of rapamycin (mTOR) signaling [15]. mTOR promotes anabolic processes such as protein synthesis to support cell proliferation and growth. It supports the production of nucleotides, lipids, and proteins necessary for cell growth [11]. Preclinical studies have shown that mTOR inhibition with rapamycin attenuates PAH development [54, 55]. Increased proliferation and migration of hPASMCs are significant contributors to obliterative pulmonary arterial remodeling and increased PVR in PAH. Recently, Shi et al. demonstrated that rapamycin inhibits viability, proliferation, and migration in hPASMCs. In this study, using the Hypoxia/Sugen rat PH model, rapamycin treatment led to notable decreases in RVSP, the Fulton index, and wall thickness in PAs [50]. Our study found that rat lungs with PAH have elevated mTOR activity.

α-Lipoic acid (α-LA) serves as a cofactor in multienzyme complexes that catalyze the oxidative decarboxylation of α-keto acids [13]. α-LA has been shown to scavenge radicals and chelate metals. As a naturally occurring antioxidant, it has been proposed as a potential therapeutic for diabetes, polyneuropathy, cataracts, ischemia/reperfusion injury, and neurodegenerative diseases [13]. Research indicates that α-LA can activate AMPK. Zhang and colleagues found that α-LA alleviated peripheral neuropathy symptoms and improved nerve function in rats with diabetic peripheral neuropathy. Overall, our results demonstrate that α-LA and AMB exert beneficial effects on pulmonary hemodynamics, RV The study indicated that α-LA reduces oxidative stress by activating AMPK, which, in turn, activates Nrf2, thereby contributing to these therapeutic benefits [14]. Accordingly, lung p-AMPK levels were higher following α-LA and AMB monotherapies and combination therapy; however, this increase reached statistical significance relative to MCT only with AMB monotherapy (*p* = 0.035), not with α-LA (*p* = 0.062) or the combination (*p* = 0.267). Therefore, the present data do not demonstrate significant AMPK activation by α-LA in this model.Histopathological evaluation of the pulmonary arteries showed antiproliferative effects with α-LA and AMB monotherapies; in contrast, the combination therapy group showed a significantly attenuated antiproliferative effect, with PCNA levels significantly higher than in either monotherapy group. These findings were accompanied by directionally consistent changes in p-AMPK levels, which were statistically significant only in the AMB group; thus, any involvement of AMPK-related signaling in the observed antiproliferative responses remains speculative. However, the comparable changes observed with combination therapy and monotherapies do not establish AMPK activation as a common underlying mechanism. The lack of an additive response may reflect overlapping or non-additive pharmacodynamic effects, although this possibility requires further investigation.

Jeon and colleagues showed that α-LA suppresses thyroid cancer cell proliferation by activating AMPK and, consequently, downregulating mTOR signaling. Therefore, it appears that α-LA indirectly inhibits mTOR in addition to activating AMPK [15]. We observed that α-LA and AMB, alone and in combination, were associated with decreased p-mTOR levels in the lung, while total mTOR levels remained unchanged. The combination treatment showed a similar effect on p-mTOR levels to the respective monotherapies. Overall, these findings suggest that changes in AMPK- and mTOR-related signaling may be associated with the observed antiproliferative effects; however, a direct mechanistic relationship cannot be established from the present data.

Inflammation is a crucial factor in PAH pathogenesis and is important for initiating and maintaining pulmonary vascular remodeling. Elevated levels of various inflammatory mediators have been reported in both PAH patients and experimental PAH models. Circulating levels of some cytokines and chemokines are abnormally elevated, contributing to excessive contractility and vascular cell proliferation. These include IL-1β, IL-6, IL-18, MCP-1, CCL5/RANTES, and others [56].

mTOR acts as an upstream regulator of NF-κB signaling, with its activation increasing NF-κB phosphorylation and promoting inflammatory gene transcription [26]. In a clinical context, NF-κB has been observed to be activated in the pulmonary vessels of patients with end-stage idiopathic PAH [57]. Experimentally, researchers reported that NF-κB, IL-6, and TNF-α levels were increased in myocardial cells of rats in an MCT-induced PAH model. Additionally, the serum levels of IL-6 and TNF-α were increased in rats with PAH [58]. Similarly, we determined that NF-κB lung levels increased in rats with PAH. This finding is consistent with histopathological data, including a high degree of lung inflammation. Additionally, our ELISA analysis showed that mTOR activation was associated with elevated NF-κB levels in rats with PAH.

Several anti-inflammatory treatments that reduce the effects of PAH have been validated in animal models [59,60]. Recent studies have shown that α-LA has anti-inflammatory effects, primarily mediated by NF-κB, which regulates inflammatory cytokines like IL-1β and IL-6 in various tissues and cell types [16,61,62,63]. In this context, Chang et al. showed that α-LA suppresses extracellular histone-induced TNF-α release, ERK/p38 MAPK activation, and NF-κB activation in macrophage cells [64]. Accordingly, we observed that treatment with α-LA, AMB, and their combination was associated with reduced pulmonary NF-κB levels. This finding was consistent with the histopathological evidence of reduced lung inflammation in the α-LA and AMB monotherapy groups; however, in the combination group, the reduction in NF-κB was not accompanied by a significant reduction in the histopathological inflammation score relative to MCT (*p* = 0.224). Although these treatments were also associated with decreased p-mTOR levels, the present findings do not establish a direct mechanistic link between mTOR and NF-κB signaling. Further mechanistic studies are therefore warranted to determine whether modulation of mTOR/NF-κB-related signaling contributes to the anti-inflammatory effects of these treatments. Zhang et al. previously demonstrated that pretreating human monocytic THP-1 cells with α-LA led to reduced NF-κB activity following LPS exposure in a dose-dependent manner. Their in vivo studies showed that α-LA decreased NF-κB activity in lung and heart tissues of LPS-induced septic mice [65].

Serum kallistatin has been proposed as a potential biomarker for diagnosis and therapeutic response in PAH. Pamukçu et al. observed that serum kallistatin levels were significantly reduced in patients with PAH [30]. Concordantly, we found that serum kallistatin levels decreased in PAH rats but increased with α-LA and/or AMB treatment. Kallistatin, a serine protease inhibitor, has vasoprotective and anti-inflammatory effects [66]. In our study, PAH was associated with reduced serum kallistatin levels and increased NF-κB levels, suggesting a possible link between kallistatin deficiency and enhanced inflammatory signaling. α-LA and/or AMB treatment increased kallistatin levels while reducing NF-κB levels, and α-LA and AMB monotherapies also reduced histopathological inflammation. These parallel changes raise the possibility that kallistatin is involved in the anti-inflammatory effects of α-LA and/or AMB; however, this remains an exploratory association rather than demonstrated mediation, and further mechanistic studies (e.g., kallistatin knockdown or supplementation) would be required before a mediating role can be established.

Evidence linking kallistatin to AMPK signaling remains limited. A recent study demonstrated that human kallistatin gene therapy improves adipose tissue remodeling and metabolic function in high-fat diet–fed mice, which was accompanied by increased p-AMPK expression in adipose tissue [67]. In our study, increased kallistatin levels were observed together with higher p-AMPK levels following α-LA and/or AMB treatment, although the p-AMPK increase was statistically significant only in the AMB group.However, there is currently no direct evidence indicating that kallistatin directly modulates AMPK signaling or that AMPK regulates kallistatin expression; nevertheless, a potential interaction between the two pathways cannot be excluded. Collectively, these findings indicate that increased kallistatin and reduced NF-κB occur in parallel following α-LA and/or AMB treatment, with significant AMPK activation observed only after AMB monotherapy; however, the present correlative data do not establish a mediating or causal role for kallistatin in AMPK activation, and this relationship should be regarded as an exploratory hypothesis for future investigation.

It should be noted that the biochemical evaluations in this study were conducted using total lung tissue homogenates to capture the overall integrated response of the pulmonary tissue in an in vivo setting. Pulmonary arterial hypertension is a multicellular disorder involving complex cross-talk between pulmonary artery smooth muscle cells (PASMCs), endothelial cells (PAECs), and infiltrating inflammatory cells. Previous studies have established that AMPK/mTOR axis dysregulation and NF-κB-mediated inflammation primarily drive proliferation and apoptosis resistance in PASMCs, alongside endothelial dysfunction in PAECs. Furthermore, Kallistatin acts as a critical protective factor targeting both endothelial integrity and vascular smooth muscle remodeling. Although our whole-tissue biochemical analysis reflects the combined tissue-level modulation of these signaling pathways, future cell-type-specific in vitro or immunohistochemical co-localization studies will be valuable to dissect the precise individual contribution of each cell population.

It should also be noted that the MCT model reproduces not only PAH-relevant vascular remodeling but also a substantial component of acute toxic and inflammatory lung injury that is not specific to pulmonary vascular disease [68]. Given the antioxidant and anti-inflammatory properties of α-LA, part of the observed benefit—particularly the reductions in pulmonary inflammatory scores and NF-κB levels—may reflect attenuation of MCT-associated toxic and inflammatory tissue injury rather than a selective reversal of PAH-specific vascular remodeling. The concurrent improvements observed in medial thickness, α-SMA, and PCNA expression provide some support for effects on vascular smooth muscle proliferation and remodeling specifically; nonetheless, the present design does not allow these two contributions to be fully disentangled.

## 5. Limitations

This study has several limitations. First, it was conducted using a monocrotaline-induced rat model, which reproduces several key inflammatory and vascular features of PAH but does not fully replicate the complex, chronic pathology of human PAH [69].

Furthermore, because α-LA has antioxidant and anti-inflammatory properties, part of the observed improvement may reflect attenuation of MCT-associated toxic and inflammatory injury rather than a selective effect on PAH-specific remodeling. This concern is partly mitigated by two observations: treatment began on day 22, after the acute MCT-pyrrole toxic/endothelial injury phase had subsided and vascular remodeling was already established, so our findings more likely reflect the reversal of established disease than prevention of acute injury; and in the combination group, remodeling parameters improved significantly without a corresponding improvement in inflammation, suggesting the anti-remodeling effect is not solely explained by reduced inflammation. Nonetheless, since inflammation persists throughout the MCT disease course, this confound cannot be fully excluded when extrapolating to human Group 1 PAH.

These findings should therefore be interpreted with caution when considering their translational relevance to human PAH. In addition, because the treatment duration was limited to 14 days, this study does not provide information on long-term treatment effects, survival, or progression to advanced right ventricular failure. Longer-term studies are needed to address these questions.

Although coordinated changes in AMPK, mTOR, NF-κB, and kallistatin were observed, these findings remain primarily associative, as no pathway-specific inhibition, activation, or genetic validation experiment was performed. A detailed molecular profile of oxidative stress was also not assessed. Further confirmation using additional experimental models and, eventually, clinical studies is therefore required before these findings can be translated into clinical practice.

An additional limitation is that biochemical signaling changes (p-AMPK, p-mTOR, NF-κB) were quantified by ELISA alone, without orthogonal validation by Western blotting for p-AMPK/AMPK and p-mTOR/mTOR ratios normalized to a loading control (e.g., β-actin or GAPDH). Although the ELISA kits used have established specificity and low intra-/inter-assay variability (Section 2.6), Western blot validation would provide complementary confirmation of these findings and is recommended for future work.

A further limitation concerns the timing of treatment initiation. α-LA and/or AMB were administered from day 22 to day 35 after MCT injection, consistent with a therapeutic rather than a preventive design; however, established pulmonary hypertension was not objectively confirmed at day 22 (e.g., by pretreatment RVSP or echocardiographic measurement in a subset of animals), so comparable disease severity across MCT-treated groups at treatment initiation was assumed based on the reproducibility of this model in prior studies rather than directly demonstrated. This should be taken into account when interpreting the magnitude of the subsequent treatment effects.

A further limitation of this study is that the biochemical analyses were performed using total lung tissue homogenates rather than isolated or cell-type-specific samples. Therefore, although the observed changes in the AMPK/mTOR/NF-κB axis and Kallistatin are consistent with mechanisms implicated in pulmonary vascular remodeling, the relative contribution of PASMCs, PAECs, and infiltrating inflammatory cells to these changes cannot be definitively determined. Future studies incorporating cell-type-specific analyses, such as immunohistochemical co-localization or isolated cell-based approaches, are warranted to clarify the cellular sources and contributions of these molecular changes.

## 6. Conclusions

Overall, our results demonstrate that α-LA and AMB exert beneficial effects on pulmonary hemodynamics, RV function, vascular proliferation, and inflammatory processes in experimental PAH, whereas a significant reduction in RV hypertrophy (Fulton index) was observed only with α-LA monotherapy. The observed antiproliferative effects may be associated with changes in AMPK- and mTOR-related signaling, whereas the anti-inflammatory effects may be associated with reduced NF-κB levels and increased kallistatin levels. Combination therapy improved RVSP and RV performance relative to MCT, and, unlike either monotherapy, α-LA + AMB did not differ significantly from the control group for RVSP, suggesting more complete normalization. However, direct statistical comparisons of α-LA + AMB versus α-LA and versus AMB monotherapy did not reach statistical significance for RVSP (*p* ≥ 0.05); therefore, although combination therapy trended toward greater improvement, formal statistical superiority over either monotherapy is not demonstrated for this endpoint. These findings suggest that changes in AMPK/mTOR/NF-κB-related signaling and kallistatin levels may contribute to the beneficial effects of α-LA and AMB in PAH; however, further mechanistic studies are needed to clarify these associations.

## Figures and Tables

**Table 1:** Animal allocation, survival, and final analyzed numbers per outcome measure and group.

Group	Initial *n*	Survived to Endpoint, *n* (%)	Echo	Histo/IHC	RVSP	Fulton	ELISA
Control	10	10 (100%)	10	10	7	10	8
MCT	12	12 (100%)	10	10	8	10	8
α-LA	12	12 (100%)	8	10	8	12	8
AMB	12	12 (100%)	9	10	7	9	8
α-LA + AMB	12	12 (100%)	10	10	9	10	8
Total	58	58 (100%)	47	50	39	51	40

**Table 2:** Outcome-specific exclusions and reasons.

Outcome	Available, *n*	Analyzed, *n*	Excluded, *n*	Reason for Exclusion	Deaths, *n*
Echocardiography	58	47	11	Inadequate acoustic window and/or image artifacts	0
Histopathology/IHC	58	50	8	Inadequate tissue preservation, sectioning artifacts, and/or poor staining quality	0
RVSP	58	39	19	Cannulation-related technical failure and/or unstable pressure tracing	0
Fulton index	58	51	7	Technical problems during ventricular separation or unreliable ventricular weight measurements	0
ELISA	58	40	18	Sample/assay quality issues, out-of-range values, or inconsistent duplicates	0

**Table 3:** Histopathological scoring system for pulmonary vascular lesions.

Parameter	Score
Small vessel obliteration and perivascular inflammation	0–2
Adventitial fibrosis in distal muscular arteries	0–1
Medial/intimal fibrosis	0–2
Interstitial fibrosis in hypoxia	0–1
Medial hypertrophy	0–2
Regulation of thrombi	0–1
Changes similar to systemic atherosclerosis are seen in large vessels/branches	0–2
Intimal remodeling and plexiform lesions	0–3

**Table 4:** Histopathological scoring of pulmonary inflammation.

Parameter	Score
Mild to moderate neutrophil infiltration (acute inflammation) is present	1
Suppurative inflammation. Degenerative neutrophils filling alveoli, necrotic debris, degenerative neutrophils foamy/active alveolar macrophages present	2
Mononuclear inflammatory cells are present, predominantly perivascular, peribronchial, and subpleural	3
Granulomatous inflammation	4

**Table 5:** Pairwise comparisons between groups for the echocardiographic parameters shown in Figure 3, including heart rate recorded during echocardiographic acquisition (Kruskal–Wallis H test: PAT, H = 21.13, *p* < 0.001; RV/LV diameter ratio, H = 25.48, *p* < 0.001; TAPSE, H = 21.59, *p* < 0.001; HR, H = 8.19, *p* = 0.085). *p* values are from Conover’s post hoc test with Holm correction; effect sizes (rank-biserial r) and 95% confidence intervals (Hodges–Lehmann median difference) were calculated independently using the Mann–Whitney U/Wilcoxon rank-sum framework (see Section 2.7). Format: *p* value (r) [95% CI].

Comparison	PAT, ms	RV/LV Diameter Ratio	TAPSE, cm	HR, bpm
AMB vs. AMB+α-LA	0.470 (−0.17) [−8.000, 4.000]	0.703 (−0.01) [−0.220, 0.180]	0.516 (−0.11) [−0.100, 0.000]	0.667 (−0.40) [−99.000, 17.000]
AMB vs. Control	0.002 (−0.70) [−15.000, −3.000]	<0.001 (0.93) [0.120, 0.310]	0.097 (−0.29) [−0.100, 0.000]	0.117 (−0.80) [−92.000, −55.000]
AMB vs. MCT	0.064 (0.42) [0.000, 8.000]	0.006 (−0.67) [−0.290, −0.050]	<0.001 (0.67) [0.000, 0.100]	1.000 (−0.28) [−56.000, 8.000]
AMB vs. α-LA	0.124 (0.36) [0.000, 8.000]	0.040 (−0.67) [−0.270, −0.030]	0.575 (0.11) [0.000, 0.100]	1.000 (0.11) [−72.000, 20.000]
AMB+α-LA vs. Control	0.013 (−0.57) [−15.000, 0.000]	<0.001 (0.78) [0.050, 0.380]	0.291 (−0.18) [−0.100, 0.000]	1.000 (−0.10) [−89.000, 30.000]
AMB+α-LA vs. MCT	0.010 (0.55) [0.000, 11.000]	0.014 (−0.47) [−0.340, 0.020]	<0.001 (0.80) [0.100, 0.100]	1.000 (0.20) [−42.000, 89.000]
AMB+α-LA vs. α-LA	0.026 (0.51) [0.000, 12.000]	0.078 (−0.32) [−0.300, 0.050]	0.231 (0.24) [0.000, 0.100]	1.000 (0.24) [−40.000, 106.000]
Control vs. MCT	<0.001 (0.98) [8.000, 19.000]	<0.001 (−1.00) [−0.470, −0.300]	<0.001 (1.00) [0.100, 0.100]	0.572 (0.57) [4.000, 88.000]
Control vs. α-LA	<0.001 (0.98) [7.000, 19.000]	<0.001 (−1.00) [−0.440, −0.290]	0.032 (0.44) [0.000, 0.100]	0.295 (0.46) [−8.000, 96.000]
MCT vs. α-LA	0.814 (−0.07) [−4.000, 4.000]	0.548 (0.23) [−0.100, 0.120]	0.003 (−0.62) [−0.100, 0.000]	1.000 (0.16) [−35.000, 52.000]

r, rank-biserial correlation (effect size); CI, confidence interval; PAT, pulmonary acceleration time; RV, right ventricle; LV, left ventricle; TAPSE, tricuspid annular plane systolic excursion; HR, heart rate recorded during echocardiographic acquisition. A negative median difference (and negative r) indicates that the first-named group has lower values than the second-named group in that comparison, and vice versa for positive values. *p* values are from Conover’s post hoc test with Holm correction; because they derive from a different framework than the Mann–Whitney U test underlying the effect size and CI columns, minor numerical discrepancies from an unadjusted Mann–Whitney *p* value may occur for individual comparisons. For HR, the omnibus Kruskal–Wallis test was not statistically significant (*p* = 0.085), so the corresponding pairwise comparisons above should be considered exploratory only.

**Table 6:** Pairwise comparisons between groups for RVSP (Figure 5) and heart rate (HR) recorded during invasive RVSP measurement (RVSP: one-way ANOVA, F = 9.47, *p* < 0.001; Levene’s test for homogeneity of variance, *p* = 0.100; HR: Kruskal–Wallis H test, H = 7.94, *p* = 0.094, omnibus not statistically significant). *p* values for RVSP are from Tukey’s HSD post hoc test; *p* values for HR are from Conover’s post hoc test with Holm correction and should be considered exploratory only, since the omnibus test for HR did not reach statistical significance. Effect sizes (rank-biserial r) and 95% confidence intervals (Hodges–Lehmann median difference) were calculated independently using the Mann–Whitney U/Wilcoxon rank-sum framework (see Section 2.7). Format: *p* value (r) [95% CI].

Comparison	RVSP, mmHg	HR, bpm
AMB vs. AMB+α-LA	0.129 (0.56) [−4.190, 33.190]	1.000 (−0.30) [−198.000, 41.000]
AMB vs. Control	0.004 (1.00) [17.810, 37.630]	0.394 (−0.71) [−139.000, −14.000]
AMB vs. MCT	0.530 (−0.32) [−23.890, 6.700]	0.993 (−0.39) [−94.000, 21.000]
AMB vs. α-LA	0.955 (0.32) [−8.980, 14.650]	1.000 (0.21) [−79.000, 85.000]
AMB+α-LA vs. Control	0.448 (0.11) [−3.840, 31.880]	1.000 (−0.24) [−112.000, 125.000]
AMB+α-LA vs. MCT	0.002 (−0.69) [−43.200, −10.380]	1.000 (−0.29) [−62.000, 67.000]
AMB+α-LA vs. α-LA	0.398 (−0.47) [−25.960, 4.610]	0.993 (0.44) [−32.000, 159.000]
Control vs. MCT	<0.001 (−1.00) [−47.830, −26.710]	1.000 (0.32) [−49.000, 95.000]
Control vs. α-LA	0.017 (−1.00) [−28.230, −16.240]	0.137 (0.64) [9.000, 159.000]
MCT vs. α-LA	0.156 (0.72) [0.830, 25.740]	0.456 (0.50) [−28.000, 115.000]

r, rank-biserial correlation (effect size); CI, confidence interval. A negative median difference (and negative r) indicates that the first-named group has a lower value than the second-named group in that comparison, and vice versa for positive values. |r| of approximately 0.1, 0.3, and 0.5 are conventionally interpreted as small, medium, and large effects, respectively. For RVSP, *p* values are from Tukey’s HSD post hoc test; because they derive from a different framework than the Mann–Whitney U test underlying the effect size and CI columns, minor numerical discrepancies from an unadjusted Mann–Whitney *p* value may occur for individual comparisons, although the direction and pattern of significance are consistent between the two approaches. Because the omnibus Kruskal–Wallis test for HR was not statistically significant, none of the HR pairwise comparisons should be considered confirmatory; they are reported for completeness and transparency only.

**Table 7:** Pairwise comparisons between groups for the Fulton index (RV/(LV + IVS)) shown in Figure 7 (Kruskal–Wallis H test, H = 10.29, *p* = 0.036). *p* values are from Conover’s post hoc test with Holm correction; effect sizes (rank-biserial r) and 95% confidence intervals (Hodges–Lehmann median difference) were calculated independently using the Mann–Whitney U/Wilcoxon rank-sum framework (see Section 2.7). Format: *p* value (r) [95% CI].

Comparison	Fulton Index, RV/(LV + IVS)
AMB vs. AMB+α-LA	0.093 (0.40) [−0.058, 0.176]
AMB vs. Control	0.012 (0.69) [0.036, 0.195]
AMB vs. MCT	0.573 (0.16) [−0.088, 0.117]
AMB vs. α-LA	0.009 (0.63) [0.038, 0.197]
AMB+α-LA vs. Control	0.361 (0.16) [−0.052, 0.133]
AMB+α-LA vs. MCT	0.243 (−0.28) [−0.159, 0.072]
AMB+α-LA vs. α-LA	0.342 (0.25) [−0.032, 0.122]
Control vs. MCT	0.041 (−0.58) [−0.183, −0.012]
Control vs. α-LA	0.998 (0.03) [−0.062, 0.066]
MCT vs. α-LA	0.033 (0.47) [−0.005, 0.182]

r, rank-biserial correlation (effect size); CI, confidence interval; RV, right ventricle; LV, left ventricle; IVS, interventricular septum. A negative median difference (and negative r) indicates that the first-named group has a lower Fulton index than the second-named group in that comparison, and vice versa for positive values. *p* values are from Conover’s post hoc test with Holm correction; because they derive from a different framework than the Mann–Whitney U test underlying the effect size and CI columns, minor numerical discrepancies from an unadjusted Mann–Whitney *p* value may occur for individual comparisons, although the direction and pattern of significance are consistent between the two approaches.

**Table 8:** Pairwise comparisons between groups for the histopathological and immunohistochemical parameters shown in Figure 9 (Kruskal–Wallis H test, all *p* < 0.001). *p* values are from Conover’s post hoc test with Holm correction; effect sizes (rank-biserial r) and 95% confidence intervals (Hodges–Lehmann median difference) were calculated independently using the Mann–Whitney U/Wilcoxon rank-sum framework (see Section 2.7). Format: *p* value (r) [95% CI].

Comparison	Media Thickness/Ext. Diameter	Mean Cardiomyocyte Diameter	Histopathological Score, Lung	Degree of Inflammation, Lung	αSMA, PAs	PCNA, PAs
AMB vs. AMB+α-LA	0.880 (0.00) [−0.036, 0.036]	0.001 (−0.72) [−2.870, −0.255]	0.075 (−0.32) [−3.0, 1.0]	0.228 (−0.27) [−1.0, 0.0]	0.024 (−0.46) [−2.0, 0.0]	0.002 (−0.64) [−1.0, 0.0]
AMB vs. Control	0.043 (0.50) [−0.001, 0.057]	<0.001 (1.00) [1.534, 3.580]	<0.001 (0.72) [0.0, 5.0]	<0.001 (0.82) [1.0, 2.0]	0.053 (0.40) [0.0, 2.0]	0.940 (−0.01) [−1.0, 0.0]
AMB vs. MCT	<0.001 (−1.00) [−0.206, −0.107]	<0.001 (−0.88) [−3.005, −0.901]	<0.001 (−0.77) [−6.0, −2.0]	0.018 (−0.49) [−2.0, 0.0]	<0.001 (−0.86) [−5.0, −2.0]	<0.001 (−0.80) [−3.0, −1.0]
AMB vs. α-LA	0.846 (−0.02) [−0.030, 0.024]	0.015 (0.60) [0.166, 1.559]	0.050 (0.33) [−1.0, 4.0]	0.878 (−0.05) [−1.0, 1.0]	0.172 (−0.26) [−2.0, 1.0]	0.839 (−0.06) [−1.0, 0.0]
AMB+α-LA vs. Control	0.060 (0.38) [−0.009, 0.063]	<0.001 (1.00) [2.440, 5.213]	<0.001 (0.98) [2.0, 5.0]	<0.001 (0.91) [1.0, 2.0]	<0.001 (0.90) [1.0, 2.0]	0.003 (0.63) [0.0, 1.0]
AMB+α-LA vs. MCT	<0.001 (−0.98) [−0.209, −0.101]	0.122 (−0.38) [−1.516, 0.985]	0.006 (−0.78) [−4.0, −1.0]	0.224 (−0.31) [−1.0, 0.0]	0.009 (−0.75) [−5.0, −2.0]	0.178 (−0.41) [−2.0, 0.0]
AMB+α-LA vs. α-LA	0.731 (−0.06) [−0.034, 0.030]	<0.001 (0.88) [0.942, 3.707]	<0.001 (0.82) [1.0, 4.0]	0.292 (0.25) [0.0, 1.0]	0.348 (0.23) [−1.0, 2.0]	0.004 (0.64) [0.0, 1.0]
Control vs. MCT	<0.001 (−0.98) [−0.224, −0.134]	<0.001 (−1.00) [−5.797, −3.295]	<0.001 (−1.00) [−8.0, −5.0]	<0.001 (−0.94) [−3.0, −1.0]	<0.001 (−0.90) [−7.0, −4.0]	<0.001 (−0.76) [−3.0, −1.0]
Control vs. α-LA	0.028 (−0.58) [−0.058, −0.004]	<0.001 (−0.84) [−2.840, −0.814]	0.029 (−0.58) [−2.0, 0.0]	<0.001 (−0.85) [−2.0, −1.0]	0.002 (−0.70) [−2.0, 0.0]	0.898 (−0.06) [−1.0, 0.0]
MCT vs. α-LA	<0.001 (1.00) [0.109, 0.192]	<0.001 (0.96) [1.809, 3.913]	<0.001 (1.00) [4.0, 7.0]	0.026 (0.50) [0.0, 2.0]	<0.001 (0.71) [2.0, 5.0]	<0.001 (0.80) [1.0, 3.0]

r, rank-biserial correlation (effect size); CI, confidence interval; PAs, pulmonary arteries. A negative median difference (and negative r) indicates that the first-named group has lower values than the second-named group in that comparison, and vice versa for positive values. |r| of approximately 0.1, 0.3, and 0.5 are conventionally interpreted as small, medium, and large effects, respectively. *p* values are those reported for the corresponding pairwise comparisons in Figure 9; because they derive from Conover’s post hoc test with Holm correction rather than the Mann–Whitney U test underlying the effect size and CI columns, minor numerical discrepancies from an unadjusted Mann–Whitney *p* value may occur for individual comparisons, although the direction and pattern of significance are consistent between the two approaches.

**Table 9:** Pairwise comparisons between groups for the ELISA parameters shown in Figure 10 (Kruskal–Wallis H test: p-mTOR, H = 15.77, *p* = 0.003; mTOR, H = 6.01, *p* = 0.199; p-AMPK, H = 13.44, *p* = 0.009; Total AMPK, one-way ANOVA F = 1.84, *p* = 0.143 (Levene’s test for homogeneity of variance, *p* = 0.157; Tukey HSD post hoc used instead of Conover–Holm for this parameter only); Kallistatin, H = 18.54, *p* < 0.001; NF-κB, H = 10.31, *p* = 0.036). *p* values are from Conover’s post hoc test with Holm correction; effect sizes (rank-biserial r) and 95% confidence intervals (Hodges–Lehmann median difference) were calculated independently using the Mann–Whitney U/Wilcoxon rank-sum framework (see Section 2.7). Format: *p* value (r) [95% CI].

Comparison	p-mTOR, ng/mL	mTOR, ng/L	p-AMPK, U/L	Total AMPK, ng/mL	Kallistatin, ng/mL	NF-κB, pg/mL
AMB vs. AMB+α-LA	0.715 (0.16) [−68.104, 58.500]	1.000 (0.38) [−15.699, 21.761]	0.864 (0.59) [−0.155, 7.054]	0.993 (0.12) [−7.781, 4.877]	0.140 (0.34) [−3.271, 10.688]	0.991 (−0.06) [−1934.190, 1289.460]
AMB vs. Control	0.032 (0.53) [−7.330, 120.201]	0.974 (−0.47) [−87.356, 20.518]	0.999 (−0.06) [−5.102, 8.584]	0.414 (0.41) [−2.731, 10.664]	0.868 (0.03) [−7.227, 7.399]	0.944 (−0.06) [−1934.190, 1289.460]
AMB vs. MCT	0.014 (−0.66) [−118.746, −15.134]	1.000 (−0.19) [−43.212, 20.518]	0.035 (1.00) [5.102, 13.543]	0.952 (−0.38) [−4.703, 1.452]	<0.001 (1.00) [6.880, 20.058]	0.006 (−0.67) [−8142.450, −214.910]
AMB vs. α-LA	0.896 (−0.06) [−35.217, 33.761]	1.000 (−0.53) [−44.766, 4.508]	0.999 (−0.06) [−7.387, 8.763]	0.999 (0.12) [−3.576, 4.335]	0.934 (0.06) [−6.094, 6.951]	0.407 (−0.22) [−2793.830, 1504.370]
AMB+α-LA vs. Control	0.071 (0.56) [−4.075, 129.170]	0.360 (−0.47) [−93.107, 14.922]	0.732 (−0.34) [−10.148, 4.881]	0.210 (0.41) [−4.140, 14.587]	0.188 (−0.38) [−11.183, 4.337]	0.953 (−0.03) [−1222.350, 1289.460]
AMB+α-LA vs. MCT	0.005 (−0.59) [−128.932, 10.395]	1.000 (−0.22) [−51.761, 16.166]	0.267 (0.75) [1.445, 11.480]	0.998 (−0.28) [−7.000, 6.134]	0.003 (0.84) [3.856, 16.297]	0.006 (−0.83) [−7306.940, −429.820]
AMB+α-LA vs. α-LA	0.621 (−0.28) [−41.911, 76.836]	0.655 (−0.59) [−50.362, 3.731]	0.947 (−0.34) [−11.310, 5.505]	0.964 (−0.06) [−5.267, 6.785]	0.163 (−0.38) [−10.626, 4.137]	0.413 (−0.20) [−2364.010, 1289.460]
Control vs. MCT	<0.001 (−0.94) [−172.589, −64.612]	1.000 (0.25) [−58.756, 83.159]	0.019 (0.69) [1.864, 26.708]	0.121 (−0.59) [−13.287, 1.084]	<0.001 (0.94) [7.275, 20.187]	0.007 (−0.80) [−7306.940, −429.820]
Control vs. α-LA	0.024 (−0.66) [−90.514, −11.060]	1.000 (0.25) [−33.730, 74.455]	0.987 (0.03) [−9.339, 13.865]	0.554 (−0.38) [−11.205, 3.295]	0.934 (−0.03) [−6.761, 8.027]	0.447 (−0.22) [−2302.607, 1289.460]
MCT vs. α-LA	0.019 (0.88) [39.873, 109.432]	1.000 (−0.09) [−48.030, 35.751]	0.062 (−0.66) [−18.800, −1.985]	0.877 (0.34) [−1.408, 6.394]	<0.001 (−1.00) [−18.334, −6.722]	0.045 (0.55) [−214.910, 7951.670]

r, rank-biserial correlation (effect size); CI, confidence interval; p-mTOR, phosphorylated-mechanistic target of rapamycin; mTOR, mechanistic target of rapamycin; p-AMPK, phosphorylated-AMP-activated protein kinase; NF-κB, nuclear factor kappa-B. A negative median difference (and negative r) indicates that the first-named group has lower values than the second-named group in that comparison, and vice versa for positive values. *p* values are from Conover’s post hoc test with Holm correction; because they derive from a different framework than the Mann–Whitney U test underlying the effect size and CI columns, minor numerical discrepancies from an unadjusted Mann–Whitney *p* value may occur for individual comparisons. For mTOR, the omnibus Kruskal–Wallis test was not statistically significant. Total AMPK was normally distributed in every group (Shapiro–Wilk *p* > 0.05) and had homogeneous variances (Levene’s *p* = 0.157), so *p* values for this parameter were obtained from one-way ANOVA with Tukey HSD post hoc rather than Kruskal–Wallis/Conover–Holm; its omnibus ANOVA was also not statistically significant (*p* = 0.143). For mTOR and Total AMPK, the corresponding pairwise comparisons above should therefore be considered exploratory only.

## Data Availability

The original contributions presented in this study are included in the article/Supplementary Material. Further inquiries can be directed to the corresponding author. Individual animal-level raw data underlying all figures are provided in Supplementary Tables S6–S10.

## Supplementary Materials

The following supporting information can be downloaded at: https://www.mdpi.com/article/10.3390/biom16101432/s1, Table S1. Shapiro–Wilk normality test results and test selection justification for echocardiographic parameters; Table S2. Shapiro–Wilk normality test results and test selection justification for RVSP and HR parameters; Table S3. Shapiro–Wilk normality test results and test selection justification for Fulton index; Table S4. Shapiro–Wilk normality test results and test selection justification for histopathology parameters; Table S5. Shapiro–Wilk normality test results and test selection justification for ELISA parameters; Table S6. Individual animal-level raw data for echocardiographic parameters; Table S7. Individual animal-level raw data for RVSP and heart rate (HR); Table S8. Individual animal-level raw data for the Fulton index (RV/(LV + IVS)); Table S9. Individual animal-level raw data for histopathological parameters; Table S10. Individual animal-level raw data for ELISA parameters.
