# Supplementary material for: α-Lipoic Acid, with or Without Ambrisentan, Attenuates Experimental Pulmonary Arterial Hypertension: Associations with AMPK/mTOR/NF-κB Signaling and Kallistatin
Source: Biomolecules. 2026 Oct 2;16(10):1432. doi: 10.3390/biom16101432 (PMC13702635; doi:10.3390/biom16101432)
Supplement: Supplementary file 1 [file biomolecules-16-01432-s001.zip › biomolecules-4542280-supplementary.pdf]

Supplementary Material

Table S1. Shapiro–Wilk normality test results and test selection justification for echocardiographic parameters.

Parameter	Group	n	Shapiro-Wilk (*W*)	*p*	*Selected Test*
PAT	Control	10	0.788	**0.010**	Kruskal-Wallis H test
	MCT	10	0.890	0.172	
	α-LA	8	0.931	0.522	
	AMB	9	0.750	**0.005**	
	α-LA+AMB	10	0.938	0.528	
RV/LV DIAM	Control	10	0.905	0.247	Kruskal-Wallis H test
	MCT	10	0.973	0.915	
	α-LA	8	0.871	0.153	
	AMB	9	0.882	0.167	
	α-LA+AMB	10	0.773	**0.007**	
Heart rate	Control	10	0.866	0.090	Kruskal-Wallis H test
	MCT	10	0.930	0.443	
	α-LA	8	0.774	**0.015**	
	AMB	9	0.948	0.669	
	α-LA+AMB	10	0.893	0.182	
TAPSE	Control	10	0.366	**<0.001**	Kruskal-Wallis H test
	MCT	10	1.000	1.000	
	α-LA	8	0.641	**<0.001**	
	AMB	9	0.813	**0.028**	
	α-LA+AMB	10	0.752	**0.004**	

Abbreviations: PAT, pulmonary artery acceleration time; RV/LV DIAM, right ventricular/left ventricular diameter ratio; TAPSE, tricuspid annular plane systolic excursion; MCT, monocrotaline; α-LA, α-lipoic acid; AMB, ambrisentan.**Note:** The Shapiro–Wilk test was used to assess normality within each experimental group. A *p* value >0.05 indicates no significant departure from a normal distribution.

Table S2. Shapiro–Wilk normality test results and test selection justification for RVSP and HR parameters.

Parameter	Group	n	Shapiro-Wilk (*W*)	*p*	*Selected Test*
**RVSP**	Control	7	0.905	0.365	One-way ANOVA test
	MCT	8	0.928	0.499	
	α-LA	8	0.924	0.461	
	AMB	7	0.949	0.717	
	α-LA+AMB	9	0.851	0.077	
**HR (bpm)**	Control	7	0.972	0.912	Kruskal-Wallis H test
	MCT	8	0.632	**< 0.001**	
	α-LA	8	0.901	0.294	
	AMB	7	0.909	0.387	
	α-LA+AMB	9	0.753	**0.006**	

Note: The Shapiro–Wilk test was used to assess normality within each experimental group. A *p* value >0.05 indicates no significant departure from a normal distribution. For **RVSP**, all study groups met the assumption of normal distribution (p > 0.05), justifying the use of the parametric **One-way ANOVA**. For

Heart Rate (HR), significant deviations from normal distribution were detected in the MCT ($p < 0.001$) and α-LA+AMB ($p = 0.006$) groups, justifying the application of the non-parametric **Kruskal-Wallis test**.

Table S3. Shapiro–Wilk normality test results and test selection justification for Fulton Index.

Parameter	Group	n	Shapiro-Wilk (*W*)	*p*	*Selected Test*
Fulton Index (RV/(LV+IVS))	Control	10	0.9668	0.8597	Kruskal-Wallis H test
	MCT	10	0.9492	0.6596	
	α-LA	12	0.8326	**0.0225**	
	AMB	9	0.9613	0.8118	
	α-LA+AMB	10	0.7864	**0.0099**	

Table S4. Shapiro–Wilk normality test results and test selection justification for histopathology parameters.

Parameter	Group	n	Shapiro-Wilk (*W*)	*p*	*Selected Test*
Medial Thickness / External diameter	Control	10	0.8016	**0.0152**	Kruskal-Wallis H test
	MCT	10	0.8727	0.1074	
	AMB	10	0.9575	0.7566	
	α-LA	10	0.9727	0.9145	
	α-LA+AMB	10	0.9061	0.2551	
Mean Cardiomyocyte Diameter	Control	10	0.9402	0.5551	Kruskal-Wallis H test
	MCT	10	0.8949	0.1923	
	AMB	10	0.8986	0.2114	
	α-LA	10	0.9687	0.8782	
	α-LA+AMB	10	0.7558	**0.0042**	
Histopathological Score (Lung)	Control	10	0.5093	**< 0.0001**	Kruskal-Wallis H test
	MCT	10	0.9381	0.5316	
	AMB	10	0.8781	0.1240	
	α-LA	10	0.8946	0.1910	
	α-LA+AMB	10	0.9662	0.8540	
Degree of Inflammation (Lung)	Control	10	0.5942	**< 0.0001**	Kruskal-Wallis H test
	MCT	10	0.9108	0.2869	
	AMB	10	0.7309	**0.0021**	
	α-LA	10	0.6553	**0.0003**	
	α-LA+AMB	10	0.7941	**0.0123**	
α-SMA (PAs)	Control	10	1.0000	1.0000	Kruskal-Wallis H test
	MCT	10	0.7871	**0.0101**	
	AMB	10	0.6405	**0.0002**	
	α-LA	10	0.8451	0.0508	
	α-LA+AMB	10	0.8391	**0.0430**	
PCNA (PAs)	Control	10	0.6033	**0.0001**	Kruskal-Wallis H test
	MCT	10	0.7809	**0.0085**	
	AMB	10	0.6497	**0.0002**	
	α-LA	10	0.6405	**0.0002**	
	α-LA+AMB	10	0.3657	**< 0.0001**	

Table S5. Shapiro–Wilk normality test results and test selection justification for ELISA parameters.

Parameter	Group	n	Shapiro-Wilk (W)	p	Selected Test
p-mTOR	Control	8	0.9825	0.9740	Kruskal-Wallis H test
	MCT	8	0.9766	0.9440	
	α-LA	8	0.9155	0.3941	
	AMB	8	0.6802	**0.0013**	
	α-LA+AMB	8	0.7816	**0.0181**	
mTOR	Control	8	0.9407	0.6184	Kruskal-Wallis H test
	MCT	8	0.7319	**0.0051**	
	α-LA	8	0.8859	0.2140	
	AMB	8	0.8813	0.1936	
	α-LA+AMB	8	0.8005	**0.0290**	
p-AMPK	Control	8	0.8940	0.2546	Kruskal-Wallis H test
	MCT	8	0.7475	**0.0077**	
	α-LA	8	0.9365	0.5775	
	AMB	8	0.9413	0.6235	
	α-LA+AMB	8	0.9370	0.5815	
Total AMPK	Control	8	0.9894	0.9941	One-way ANOVA test
	MCT	8	0.9599	0.8090	
	α-LA	8	0.9070	0.3336	
	AMB	8	0.9409	0.6195	
	α-LA+AMB	8	0.8425	0.0799	
Kallistatin	Control	8	0.8942	0.2562	Kruskal-Wallis H test
	MCT	8	0.8790	0.1843	
	α-LA	8	0.9061	0.3272	
	AMB	8	0.9041	0.3141	
	α-LA+AMB	8	0.7696	**0.0134**	
NF-κB	Control	8	0.9782	0.9536	Kruskal-Wallis H test
	MCT	8	0.7286	**0.0047**	
	α-LA	8	0.9311	0.5257	
	AMB	8	0.9480	0.6906	
	α-LA+AMB	8	0.9507	0.7178	

Violation of Normality: Except for **Total AMPK**, at least one experimental group in all parameters violated the assumption of normal distribution ($p \leq 0.05$).

Statistical Test Selection:.

- When the normality assumption is violated in any group within a parameter, the non-parametric **Kruskal-Wallis** test is recommended for overall group comparisons.

- The parametric **One-way ANOVA** test is appropriate only when data across all experimental groups are normally distributed ($p > 0.05$), as observed in the **Total AMPK** parameter.

Supplementary Raw Data. Individual animal-level numerical data underlying the figures

The following tables (Tables S6-S10) present the individual, animal-level raw numerical data used to generate the group means and statistical comparisons shown in the main figures, in response to the reviewer's request for transparency regarding data variability and potential outliers.

Table S6. Individual animal-level raw data for echocardiographic parameters.

Group	PAT (ms)	RV Diameter (mm)	LV Diameter (mm)	RV/LV Diameter	Heart Rate (bpm)	TAPSE (cm)
Control	30.0	3.74	5.66	0.66	310	0.2
Control	41	3.83	5.97	0.64	290	0.2
Control	42	3.84	5.14	0.74	211	0.2
Control	33	3.32	5.58	0.59	338	0.2
Control	59	4.32	6.47	0.66	310	0.2
Control	37	3.28	5.71	0.57	318	0.3
Control	33	3.61	6.35	0.56	301	0.2
Control	37	3.11	5.04	0.61	325	0.2
Control	33	3.67	6.1	0.6	331	0.2
Control	30	3.69	6.35	0.58	369	0.2
MCT	22	5.05	4.89	1.03	216	0.1
MCT	26	4.16	5.1	0.81	258	0.1
MCT	22	5.3	5.76	0.92	219	0.1
MCT	26	4.91	5.15	0.95	242	0.1
MCT	22	4.29	3.7	1.15	250	0.1
MCT	18	3.96	3.53	1.12	271	0.1
MCT	22	4.9	4.54	1.07	346	0.1
MCT	30	4.68	4.5	1.04	301	0.1
MCT	22	5.01	5.38	0.93	242	0.1
MCT	26	4.72	4.64	1.01	306	0.1
α-LA	26	4.02	4.32	0.93	318	0.1
α-LA	22	5.11	4.33	1.18	216	0.1
α-LA	26	5.28	5.73	0.92	225	0.2
α-LA	26	4.21	4.61	0.91	377	0.2
α-LA	18	5.38	5.21	1.03	242	0.1
α-LA	22	5.32	5.97	0.89	254	0.2
α-LA	30	5.41	5.43	0.99	229	0.2
α-LA	22	5.11	4.92	1.03	229	0.2
AMB	22	5.04	5	1	246	0.1
AMB	22	5.33	4.81	1.1	246	0.1
AMB	26	4.69	6.15	0.76	235	0.2
AMB	33	3.82	5.22	0.73	250	0.2
AMB	52	3.67	5.05	0.72	246	0.1
AMB	30	4.33	5.97	0.72	235	0.2
AMB	26	5.21	5.97	0.87	243	0.3
AMB	29	4.79	5.42	0.88	222	0.2
AMB	22	4.61	5.45	0.85	262	0.2
α-LA+AMB	18	4.56	4.8	0.95	331	0.2
α-LA+AMB	22	7.31	4.19	1.74	229	0.1
α-LA+AMB	41	3.86	6.07	0.63	285	0.2
α-LA+AMB	30	5.38	5.26	1.02	189	0.2
α-LA+AMB	33	4.24	6.35	0.66	187	0.2
α-LA+AMB	26	3.84	4.26	0.9	345	0.2
α-LA+AMB	30	4.78	4.76	1	331	0.2
α-LA+AMB	41	3.8	5.64	0.67	254	0.3
α-LA+AMB	30	3.11	4.92	0.63	338	0.2
α-LA+AMB	26	5.32	5.63	0.94	369	0.1

Abbreviations: PAT, pulmonary artery acceleration time; RV/LV Diameter, right ventricular/left ventricular diameter ratio; TAPSE, tricuspid annular plane systolic excursion; MCT, monocrotaline; α-LA, α-lipoic acid; AMB, ambrisentan.

Table S7. Individual animal-level raw data for RVSP and heart rate (HR).

Group	RVSP (mmHg)	HR (bpm)
Control	34.26	336
Control	31.3	202
Control	31.65	323
Control	33.02	289
Control	32.13	405
Control	33.95	230
Control	33.83	291
MCT	50.09	286
MCT	63.09	228
MCT	63.06	490
MCT	93.45	251
MCT	61.96	220
MCT	59.73	260
MCT	74.36	246
MCT	80.85	250
α-LA	58.5	248
α-LA	51.15	354
α-LA	62.64	193
α-LA	55.11	136
α-LA	38.75	172
α-LA	55.14	139
α-LA	49.26	177
α-LA	61.25	280
AMB	60.23	208
AMB	69.76	186
AMB	52.27	197
AMB	39.17	257
AMB	71.87	157
AMB	51.76	275
AMB	63.89	271
α-LA+AMB	49.35	469
α-LA+AMB	65.83	224
α-LA+AMB	29.18	235
α-LA+AMB	77.58	220
α-LA+AMB	30.11	216
α-LA+AMB	36.57	179
α-LA+AMB	31.16	530
α-LA+AMB	27.09	207
α-LA+AMB	43.36	295

Abbreviations: RVSP, right ventricular systolic pressure; HR, heart rate; MCT, monocrotaline; α-LA, α-lipoic acid; AMB, ambrisentan.

Table S8. Individual animal-level raw data for the Fulton Index (RV/(LV+IVS)).

Group	RV/(LV+IVS)
Control	0.3636
Control	0.2833
Control	0.3065
Control	0.3594
Control	0.4516
Control	0.2456
Control	0.3065
Control	0.3774
Control	0.2308
Control	0.3913
MCT	0.3898
MCT	0.4898
MCT	0.2951
MCT	0.4545
MCT	0.4058
MCT	0.2353
MCT	0.5532
MCT	0.5614
MCT	0.4082
MCT	0.4507
α-LA	0.3263
α-LA	0.3607
α-LA	0.4085
α-LA	0.2586
α-LA	0.3004
α-LA	0.2404
α-LA	0.2722
α-LA	0.3172
α-LA	0.5695
α-LA	0.3141
α-LA	0.3681
α-LA	0.3021
AMB	0.4769
AMB	0.5682
AMB	0.3014
AMB	0.3553
AMB	0.4375
AMB	0.4677
AMB	0.4
AMB	0.5588
AMB	0.4559
α-LA+AMB	0.2917
α-LA+AMB	0.6167
α-LA+AMB	0.7031
α-LA+AMB	0.2771
α-LA+AMB	0.4308
α-LA+AMB	0.3068
α-LA+AMB	0.3636
α-LA+AMB	0.2824
α-LA+AMB	0.339
α-LA+AMB	0.3623

Abbreviations: RV, right ventricle; LV, left ventricle; IVS, interventricular septum; MCT, monocrotaline; α-LA, α-lipoic acid; AMB, ambrisentan.

Table S9. Individual animal-level raw data for histopathological parameters.

Group	Medial Thickness/External Diameter (PAs)	Mean Cardiomyocyte Diameter (μm)	Histopathological Score (Lung)	Degree of Inflammation (Lung)	α-SMA (PAs)	PCNA (PAs)
Control	0.1227	5.909	0	0	2	1
Control	0.1597	5.209	0	1	2	2
Control	0.1651	5.422	0	0	2	1
Control	0.1324	7.382	0	0	2	1
Control	0.1609	7.953	1	0	2	1
Control	0.2528	6.619	0	1	2	1
Control	0.1329	8.551	0	0	2	1
Control	0.1224	7.255	1	0	2	1
Control	0.1564	7.253	0	1	2	4
Control	0.175	7.222	0	0	2	2
MCT	0.2994	10.005	8	2	6	4
MCT	0.2809	11.218	5	2	2	4
MCT	0.3208	9.826	5	3	9	2
MCT	0.2439	13.346	5	3	6	2
MCT	0.477	12.48	8	1	9	2
MCT	0.3279	10.834	4	1	9	2
MCT	0.3556	10.677	7	4	6	6
MCT	0.3558	13.619	9	3	6	4
MCT	0.3121	11.119	6	2	6	2
MCT	0.515	11.006	7	2	6	4
AMB	0.188	9.641	6	2	2	1
AMB	0.1796	8.669	5	2	4	2
AMB	0.1494	9.604	1	3	2	1
AMB	0.2066	9.331	0	1	2	1
AMB	0.1735	10.341	2	1	2	1
AMB	0.1811	9.103	6	1	4	1
AMB	0.1877	9.255	0	1	4	2
AMB	0.1217	8.673	3	1	2	3
AMB	0.1979	10.96	2	1	2	1
AMB	0.2425	8.788	1	2	4	1
α-LA	0.2281	8.196	3	2	6	1
α-LA	0.19	7.587	1	2	2	2
α-LA	0.1691	7.306	0	1	3	2
α-LA	0.1705	8.783	2	2	6	2
α-LA	0.2139	10.159	1	1	4	1
α-LA	0.1313	8.397	2	2	2	1
α-LA	0.1907	8.791	2	1	3	2
α-LA	0.1974	8.261	0	1	4	1
α-LA	0.1862	8.982	1	2	2	1
α-LA	0.1641	9.166	0	1	3	1
α-LA+AMB	0.1522	9.693	5	2	3	2
α-LA+AMB	0.2154	9.741	4	3	3	4
α-LA+AMB	0.1438	9.689	2	1	4	2
α-LA+AMB	0.1613	9.924	3	2	4	2
α-LA+AMB	0.1594	9.51	6	1	4	2
α-LA+AMB	0.1467	9.755	4	1	2	2
α-LA+AMB	0.1959	12.595	5	2	4	2
α-LA+AMB	0.194	12.103	3	2	4	2
α-LA+AMB	0.247	11.973	1	2	4	2
α-LA+AMB	0.2198	12.706	4	2	6	2

Abbreviations: PAs, pulmonary arteries; α-SMA, alpha-smooth muscle actin; PCNA, proliferating cell nuclear antigen; MCT, monocrotaline; α-LA, α-lipoic acid; AMB, ambrisentan.

Table S10. Individual animal-level raw data for ELISA parameters.

Group	p-mTOR	mTOR	p-AMPK	Total AMPK	Kallistatin	NF-κB
Control	199.626	375.253	68.523	33.971	62.099	1757.29
Control	250.85	342.455	51.62	30.134	47.426	2187.11
Control	266.857	338.569	49.002	26.363	45.336	3046.75
Control	305.275	318.518	45.031	23.047	43.617	3476.57
Control	328.559	312.611	37.378	20.511	41.437	3691.48
Control	382.984	274.84	35.094	19.297	38.932	4121.3
Control	289.03	237.379	47.77	17.021	37.076	4980.94
Control	289.03	224.944	47.77	11.212	33.681	3323.063
MCT	466.804	473.956	17.436	34.664	23.897	22757.2
MCT	439.446	298.311	33.23	31.24	25.778	10353.69
MCT	432.17	296.135	40.307	30.308	25.783	10138.78
MCT	405.103	286.809	40.462	29.05	27.239	5840.58
MCT	400.446	273.13	41.371	28.4	31.482	4336.21
MCT	369.887	247.794	41.815	28.313	32.677	4121.3
MCT	340.782	240.799	35.77	27.707	33.8	4121.3
MCT	407.805	229.452	35.77	24.737	34.162	3691.48
α-LA	316.335	346.341	56.7	31.716	49.216	1972.2
α-LA	316.917	300.798	54.57	29.874	48.721	2187.11
α-LA	324.193	294.736	50.4	28.964	45.426	2402.02
α-LA	330.014	278.726	46.85	28.639	44.112	3691.48
α-LA	341.364	269.866	46.717	26.991	40.442	3906.39
α-LA	343.111	262.094	45.209	23.068	39.399	4551.12
α-LA	356.499	260.384	37.755	23.025	37.004	5625.67
α-LA	359.118	251.213	29.37	21.919	36.914	6915.13
AMB	281.7	276.705	49.313	31.001	52.735	1112.56
AMB	302.656	274.374	48.98	29.961	48.55	1112.56
AMB	319.245	272.975	48.603	29.159	46.35	2211.24
AMB	325.648	258.519	47.095	27.403	43.122	2831.84
AMB	328.85	257.897	46.518	26.298	40.68	3476.57
AMB	350.096	255.099	45.409	25.951	38.39	4121.3
AMB	371.051	249.97	45.076	24.976	38.109	4336.21
AMB	599.812	247.949	43.678	24.39	37.642	6270.4
α-LA+AMB	291.596	308.104	50.311	44.721	63.742	1972.2
α-LA+AMB	296.253	274.218	47.25	35.184	43.536	2254.22
α-LA+AMB	301.2	256.965	42.259	28.704	41.38	3046.75
α-LA+AMB	306.73	254.944	38.842	25.994	38.704	3261.66
α-LA+AMB	325.066	249.348	37.622	25.539	38.018	3261.66
α-LA+AMB	337.872	246.239	43.26	25.084	34.533	3691.48
α-LA+AMB	418.2	244.374	43.26	21.724	33.486	3906.39
α-LA+AMB	484.849	239.866	43.26	21.313	32.434	5195.85

Abbreviations: mTOR, mechanistic target of rapamycin; AMPK, AMP-activated protein kinase; NF-κB, nuclear factor kappa B; MCT, monocrotaline; α-LA, α-lipoic acid; AMB, ambrisentan.
